# Synthesis of cobalt tetra-2,3-pyridiniumporphyrazinato with sulfonic acid tags as an efficient catalyst and its application for the synthesis of bicyclic *ortho*-aminocarbonitriles, cyclohexa-1,3-dienamines and 2-amino-3-cyanopyridines†

**DOI:** 10.1039/d0ra02172e

**Published:** 2020-07-24

**Authors:** Mohammad Dashteh, Mohammad Ali Zolfigol, Ardeshir Khazaei, Saeed Baghery, Meysam Yarie, Sajjad Makhdoomi, Maliheh Safaiee

**Affiliations:** Department of Organic Chemistry, Faculty of Chemistry, Bu-Ali Sina University Hamedan 6517838683 Iran zolfi@basu.ac.ir mzolfigol@yahoo.com Khazaei_1326@yahoo.com saadybaghery@yahoo.com +988133493009; Department of Pharmacology and Toxicology, School of Pharmacy, Hamedan University of Medicinal Science Hamedan Iran; Department of Medicinal Plants Production, University of Nahavand Nahavand 6593139565 Iran azalia_s@yahoo.com

## Abstract

Cobalt tetra-2,3-pyridiniumporphyrazinato with sulfonic acid tag [Co(TPPASO_3_H)]Cl was produced and catalyzed the synthesis of *ortho*-aminocarbonitriles, cyclohexa-1,3-dienamines and 2-amino-3-cyanopyridines. The synthesis of 2-amino-3-cyanopyridines by using [Co(TPPASO_3_H)]Cl proceeded *via* a cooperative vinylogous anomeric based oxidation mechanism. [Co(TPPASO_3_H)]Cl can be recycled and reused six times with a marginal decreasing of its catalytic activity.

## Abstract 2

*ortho*-Aminocarbonitriles, cyclohexa-1,3-dienamines and 2-amino-3-cyanopyridines were synthesized by [Co(TPPASO_3_H)]Cl as an efficient pyridiniumporphyrazinato based catalyst.

## Graphical abstract

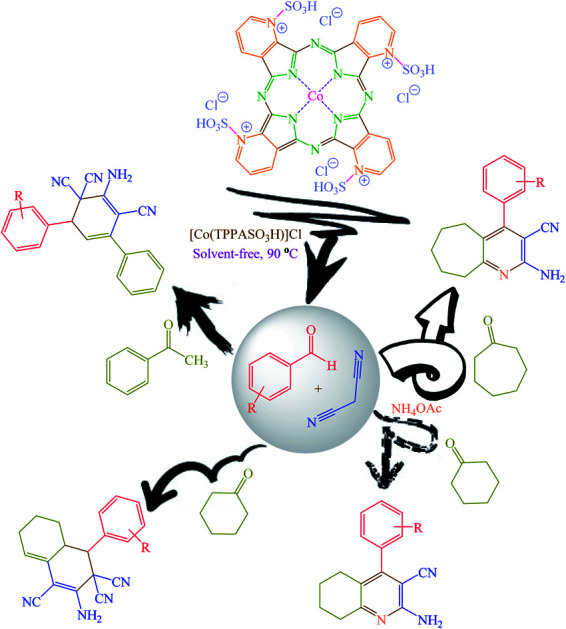

## Introduction

Tetrapyridinoporphyrazines and phthalocyanines, as high temperature materials, have various potential applications for electronic, adhesive and structural uses.^1–3^ Also, the most significant feature which makes these molecules play an excellent role in the area of materials science is their usefulness.^4^ These compounds metal complexes have been known as catalysts for several chemical reactions^5–7^ where the metal of the macro-cyclic complex acts as a redox center.

*ortho*-Aminocarbonitriles and cyclohexa-1,3-dienamines are useful precursors for the synthesis of their respective dicyanoanilines^8^ which are important for their optical properties. These compounds are possibly important skeleton in organic synthesis^9,10^ and broadly used in the synthesis of several heterocyclic compounds.^11,12^ Numerous catalysts or reagents have been used for the synthesis of these compounds include DABCO functionalized dicationic ionic liquid,^13^ borax,^14^*ortho*-benzenedisulfonimide (OBS) and triethylammonium acetate,^15^ imidazole^16^ and DES.^17^

2-Amino-3-cyanopyridines are significant compounds due to their biological and pharmaceutical activities include anti-tumor properties,^18^ anti-parkinsonism,^19^ cardiotonic^20^ and anti-inflammatory.^21,22^ A wide range of approaches for the synthesis of these compounds are being reported in the literature by numerous catalysts such as Fe_3_O_4_@niacin,^23^ Fe_3_O_4_/cellulose nanocomposite^24^ and Yb(PFO)_3_.^25^

**Scheme 1:**
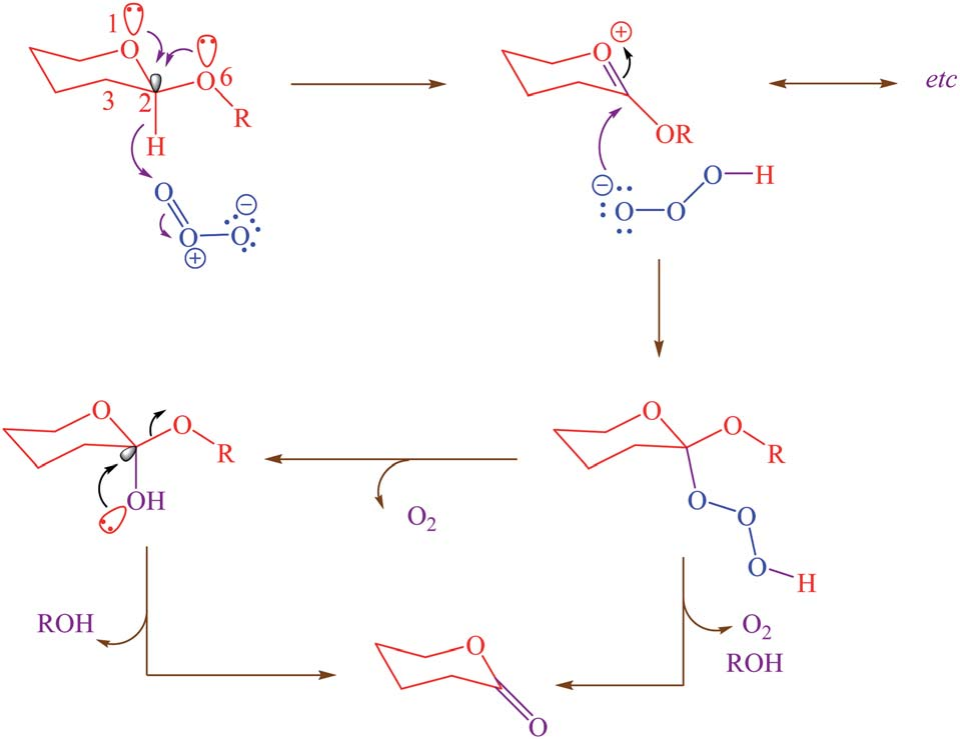
Cooperative anomeric effect in the reaction of the β-anomer of cyclic acetal.

On the other hand, the role of negative hyperconjugation in a classic conformational preference, which has been named anomeric effect, is a paramount important on the structure and reactivity of a wide variety of organic functional groups. For example, cooperative anomeric effect (more than one anomeric effect acts simultaneously) can explains some unexpected obtained experimental results. This stereo electronic interaction occurred when more than one donor and an acceptor exist in a single molecule such as cyclic acetals 1,4-dihydropyridines and *etc.* The reaction of ozone with acetals for correlating the reactivity with conformation and structure had been investigated.^26^ The reported results had been showed that the β-anomer of cyclic acetals reacted with ozone faster than α-anomer. Exo and endo anomeric effects within the β-anomer of cyclic acetals cooperatively participate in the course of reaction *via* electron donating and supporting with two anomeric effects (Scheme 1).

**Scheme 2:**
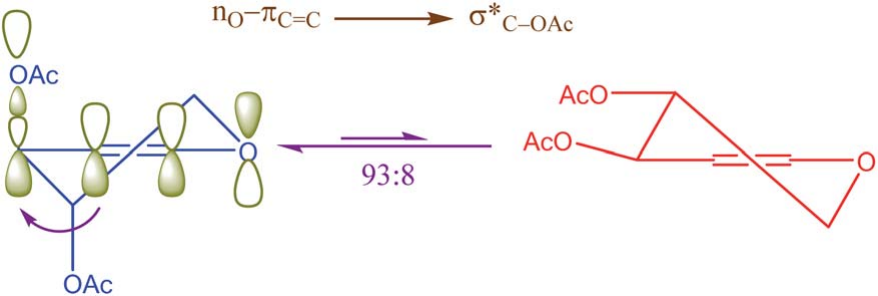
Vinylogous anomeric effect (VAE) or allylic effect in glycal.

Another kind of anomeric effect is vinylogous anomeric effect. The anomeric effect can be prolonged through double bonds which has been named vinylogous anomeric effect (VAE) or allylic effect (Scheme 2). This effect initially defined by Ferrier and Sankey as the allylic effect,^27^ which applied for explanation of stabilized pseudoaxial orientation of the acyloxy group at C-3 in a glycal. VAE can contend effectively with the gauche effect but is invalidate when the gauche effect is facilitated by additional axial alkyl substituents.^28^ This orbital interaction leads to C3–O bond lengthening and improves reactivity. It can be described in term of the stabilizing hyperconjugative interaction between the lone pair of endocyclic oxygen and the C3–O antibonding orbital, mediated by the relay alkene (the higher energy combination of lone pair and π_C=C_).^28^

**Scheme 3:**
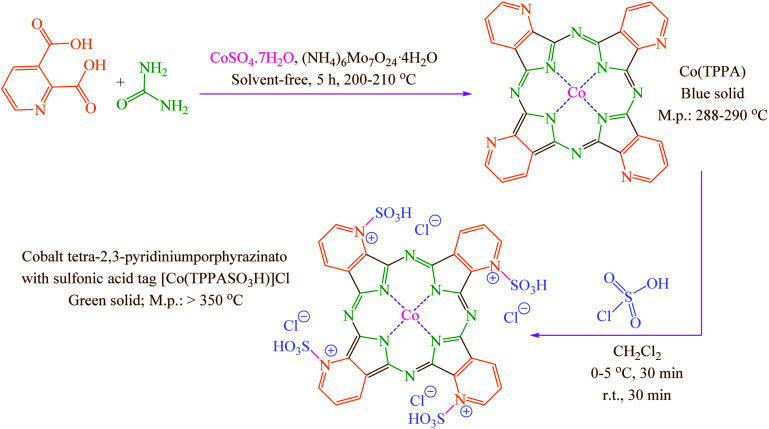
Synthesis of cobalt tetra-2,3-pyridiniumporphyrazinato with sulfonic acid tags [Co(TPPASO_3_H)]Cl.

**Scheme 4:**
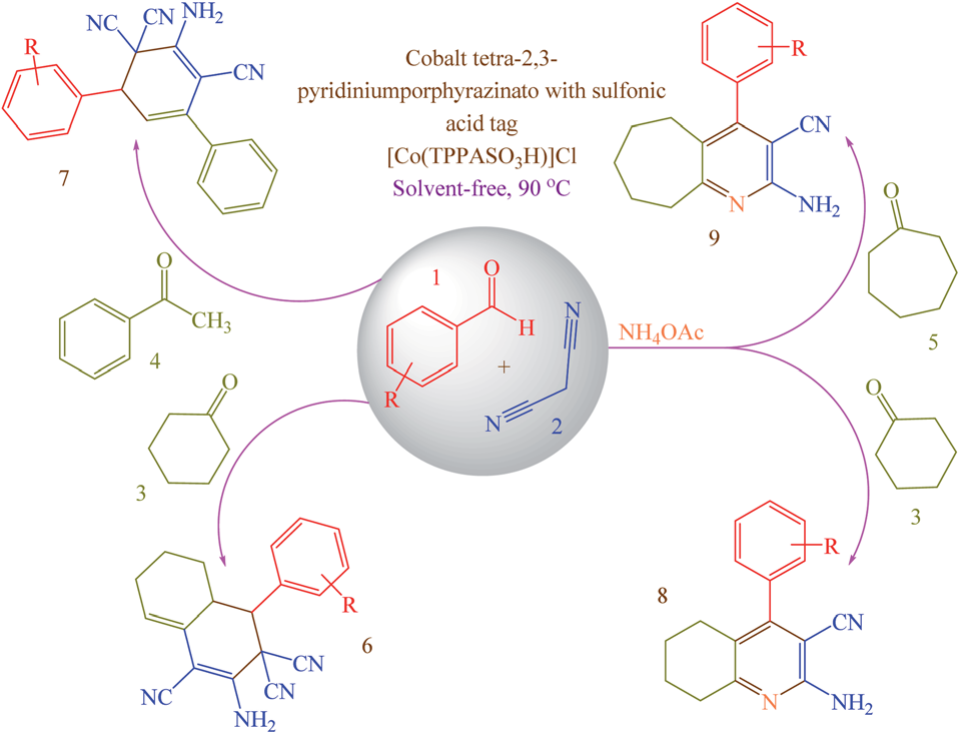
Synthesis of *ortho*-aminocarbonitriles (6), cyclohexa-1,3-dienamines (7) and 2-amino-3-cyanopyridines (8 and 9) using [Co(TPPASO_3_H)]Cl.

Concerning the mentioned points and the authors' research on the phthalocyanines-based catalysts and multi-component reactions and applications of novel catalysts,^29,30^ in this study cobalt tetra-2,3-pyridiniumporphyrazinato with sulfonic acid tags [Co(TPPASO_3_H)]Cl was synthesized (Scheme 3). Then, its catalytic activity was evaluated by multi-component reaction for the synthesis of *ortho*-aminocarbonitriles, (6) cyclohexa-1,3-dienamines (7) and 2-amino-3-cyanopyridines (8 and 9) (Scheme 4).

## Experimental

### General procedure for the synthesis [Co(TPPASO_3_H)]Cl

Co(TPPA) was synthesized according to the reported procedure.^31^ To a round-bottomed flask (50 mL) containing a solution of Co(TPPA) (8.564 mg; according to cobalt content determined by ICP analysis) in CH_2_Cl_2_ (20 mL), ClSO_3_H (4 mmol; 0.466 mg; 0.266 mL) were added over a period of 30 min whereas stirring and cooling to keep the temperature at 0–5 °C. Then, the reaction mixture was stirred for a further period of 30 min at room temperature. The attained green solid [Co(TPPASO_3_H)]Cl was washed three times with diethyl ether, dichloromethane and then dried under vacuum (isolated yield 79%; 0.375 mg) (Scheme 3).

### General procedure for the synthesis of products 6–9

[Co(TPPASO_3_H)]Cl (1 mg) as a catalyst was added to a mixture of aldehyde (1 mmol), malononitrile (2 mmol for the synthesis of 6 or 7; 1 mmol for the synthesis of 8 or 9) and cyclohexanone (1 mmol for the synthesis of 6 or 8) or acetophenone (1 mmol for the synthesis of 7) or cycloheptanone (1 mmol for the synthesis of 9) under solvent-free conditions at 90 °C (Table 2). At the end of reaction which was monitored by TLC (*n*-hexane/ethyl acetate: 5/2), the resulting mixture was washed with ethanol and filtered to separate catalyst from other materials (the catalyst was insoluble in ethanol and reaction mixture was soluble). The solvent was removed and the crude product was purified by recrystallization from ethanol to yield pure products.

## Results and discussion

### Characterization of cobalt tetra-2,3-pyridiniumporphyrazinato with sulfonic acid tag [Co(TPPASO_3_H)]Cl

Co(TPPA)^31^ was synthesized using treat between urea, 2,3-pyridine-dicarboxylic acid, (NH_4_)_6_Mo_7_O_24_·4H_2_O and CoSO_4_·7H_2_O. Then [Co(TPPASO_3_H)]Cl was produced by reaction between Co(TPPA) and ClSO_3_H in CH_2_Cl_2_. Characterization of [Co(TPPASO_3_H)]Cl was investigated by ICP, UV-vis, FT-IR, TGA, DTA, EDX, SEM coupled EDX (SEM mapping), XRD, FE-SEM, TEM and DRS.

#### ICP analysis

ICP analysis of [Co(TPPASO_3_H)]Cl and Co(TPPA)^26^ was studied for the determination of cobalt content, which presented values of 6.388 mg L^−1^ and 8.564 mg L^−1^, respectively.

#### UV-vis analysis

**Fig. 1:**
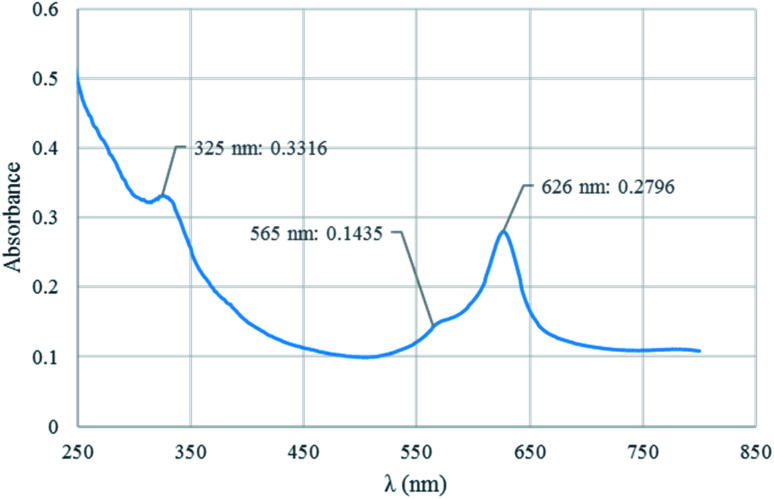
UV-vis spectrum of [Co(TPPASO_3_H)]Cl.

The structure of Co(TPPA) and [Co(TPPASO_3_H)]Cl was investigated by UV-vis spectroscopy in ethanol solvent (Fig. 1). UV-vis analysis of [Co(TPPASO_3_H)]Cl was showed three absorption peaks at 626 nm (related to Q band or blue-shifted), 565 nm (linked to charge transfer transitions between Q and B band) and 365 nm (connected to B or Soret band). Also the related absorbance is decreased. The optical band-gap energy (*E*_bg_ = 1240/*λ*_max_) for [Co(TPPASO_3_H)]Cl was calculated, which resulted value of 1.98 eV. The *λ*_max_ and absorbance changes of [Co(TPPASO_3_H)]Cl in comparison with Co(TPPA)^26^ presented synthesis of [Co(TPPASO_3_H)]Cl.

#### FT-IR analysis

**Fig. 2:**
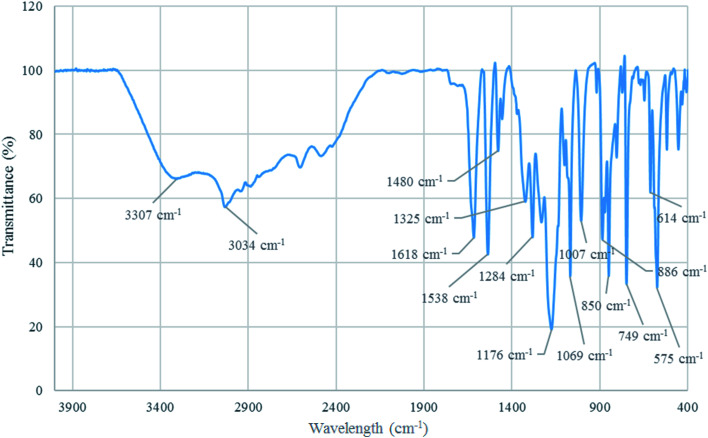
FT-IR spectrum of [Co(TPPASO_3_H)]Cl.

The structure of [Co(TPPASO_3_H)]Cl was investigated by FT-IR spectroscopy. The absorption bands at 1618 cm^−1^ and 1480 cm^−1^ connected to C=N and C=C stretching. Additionally, the absorption band at 3307 cm^−1^ related to –OH stretching in sulfonic acid tag. Also, the absorption bands at 1284 cm^−1^ and 1176 cm^−1^ linked to S=O stretching in sulfonic acid tag. The absorption band at 1069 cm^−1^ connected to S–O vibrational modes of sulfonic acid tag. Moreover, the absorption band at 575 cm^−1^ related to the cobalt-ligand stretching vibrational modes. The wavelength changes of [Co(TPPASO_3_H)]Cl in comparison with Co(TPPA) and other substrates^26^ showed synthesis of [Co(TPPASO_3_H)]Cl (Fig. 2).

#### TGA and DTA analysis

**Fig. 3:**
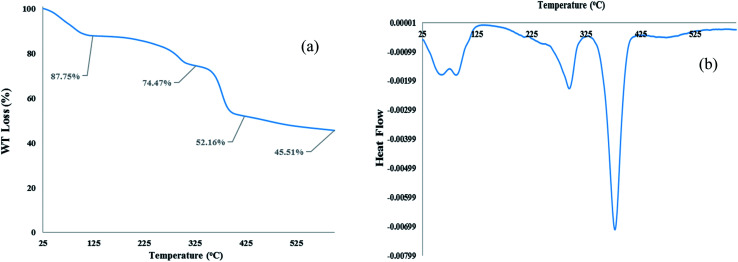
TGA (a) and DTA (b) analysis of [Co(TPPASO_3_H)]Cl.

TGA and DTA analysis of [Co(TPPASO_3_H)]Cl were studied, which showed important decrease in three steps, and decomposed above 378 °C (Fig. 3). The weight loss (about 12.25%) around 25–100 °C linked to the removal of surface-adsorbed solvent in the course of the synthesis of this compound. The next weight loss about (13.28%) up to 294 °C was may be owing to decomposition of sulfonic acid tag. The last weight loss (about 22.31%) up to 378 °C is related to the decomposition of [Co(TPPASO_3_H)]Cl. TGA and DTA analysis shows that this compound is stable up to 378 °C. The DTA analysis diagram shows a negative downward slope. Furthermore, decomposition of [Co(TPPASO_3_H)]Cl was exothermic. Thermal analysis changes of [Co(TPPASO_3_H)]Cl in comparison with Co(TPPA)^26^ confirmed its synthesis.

#### EDX analysis and SEM mapping

**Fig. 4:**
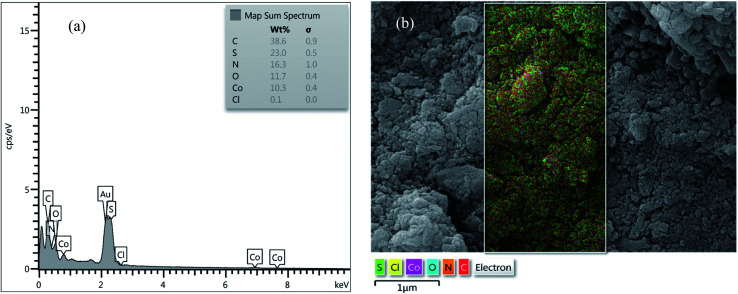
EDX analysis (a) and SEM coupled EDX (SEM mapping) (b) of [Co(TPPASO_3_H)]Cl.

EDX analysis of [Co(TPPASO_3_H)]Cl and Co(TPPA) were investigated, which clearly represents the appearance of C (38.6%), S (23.0%), N (16.3%), O (11.7%), Co (10.3%) and Cl (0.1%) signals in the structure of [Co(TPPASO_3_H)]Cl (Fig. 4). No extra impurity peaks were known in the SEM coupled EDX. The SEM coupled EDX analysis changes of [Co(TPPASO_3_H)]Cl in comparison with Co(TPPA)^26^ presented formation of its.

#### XRD, FE-SEM and TEM analysis

**Fig. 5:**
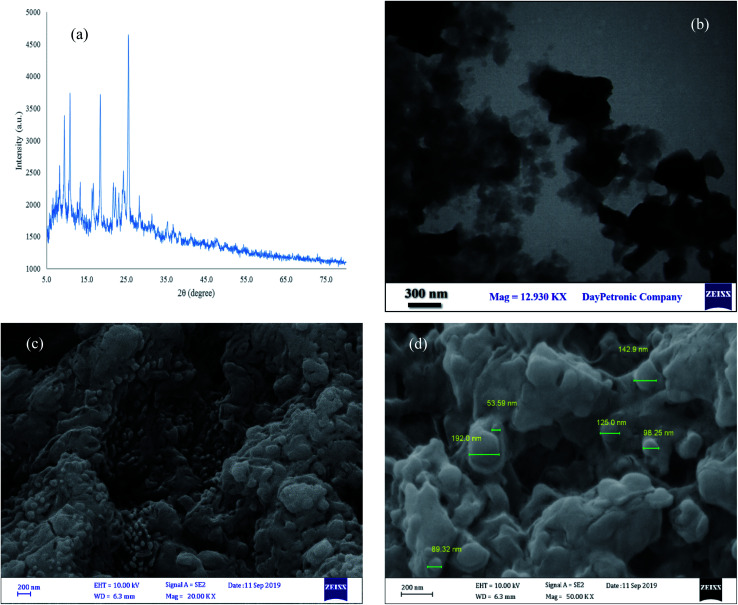
XRD pattern (a), TEM (b) and SEM (c and d) analysis of [Co(TPPASO_3_H)]Cl.

The structure of [Co(TPPASO_3_H)]Cl was studied by FE-SEM, TEM and XRD analysis (Fig. 5). By using these analyses the particle size, morphology and shape of [Co(TPPASO_3_H)]Cl were studied. According to FE-SEM and TEM images of [Co(TPPASO_3_H)]Cl, the morphology of this compound is fibrillar and its was synthesized with suitable monodispersity. The TEM micrograph of [Co(TPPASO_3_H)]Cl afforded its distribution and morphology. XRD analysis of [Co(TPPASO_3_H)]Cl was approved in solid state form with four intense peaks in 2*θ* values at 9.38°, 10.78°, 18.40° and 25.39°. The prolonged peak shows the its crystallite structure. The XRD pattern changes of [Co(TPPASO_3_H)]Cl in comparison with Co(TPPA)^26^ displayed synthesis of its. The results of XRD, FE-SEM and TEM analysis of [Co(TPPASO_3_H)]Cl displays that particle size of its above 100 nm. Additionally, the XRD pattern changes of [Co(TPPASO_3_H)]Cl in comparison with Co(TPPA)^26^ confirmed its synthesis.

#### DRS analysis

**Fig. 6:**
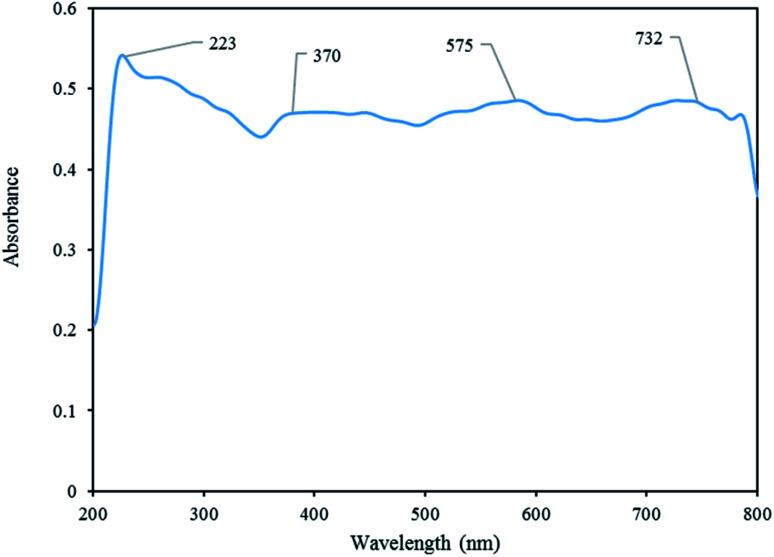
DRS analysis of [Co(TPPASO_3_H)]Cl.

The optical properties of [Co(TPPASO_3_H)]Cl was investigated by DRS analysis. The broad band at around 732 nm is attributed to the electronic ligand-field transition of Co^2+^ in tetrahedral coordination. The band at 223 nm is ascribed to Co^2+^ interacting with oxygen atoms in the SO_4_^2−^ and H_2_O structure and the band between 370 nm and 575 nm to be attributed Co^2+^ in octahedral coordination (Fig. 6).^32^

### Catalytic application of [Co(TPPASO_3_H)]Cl in the synthesis of target molecules

To investigation the catalytic activity of [Co(TPPASO_3_H)]Cl and to determine the possibility of the transformation, reaction between benzaldehyde, cyclohexanone and malononitrile for the synthesis of 6a (molar ratio: 1 : 1 : 2) was chosen as a model to optimize the reaction conditions (Table 1). Initially, we performed reaction at 90 °C under catalyst-free and solvent-free conditions, but after a prolonged time for 180 min no synthesis of 6a was detected (Table 1, entry 1). The reaction completed effectively with an appropriate yield of 95% by using 1 mg of catalyst under solvent-free conditions (Table 1, entry 3). Increasing the amount of [Co(TPPASO_3_H)]Cl to 2 mg (Table 1, entry 5) displayed no significant progress in the yield, while the yield decreased *via* decreasing the amount of the catalyst to 0.5 mg (Table 1, entry 2). It was found that the reaction could not progress efficiently in organic solvent and water except by using [Co(TPPASO_3_H)]Cl (Table 1, entries 10–16). We observed that, the 6a was not synthesized in CHCl_3_ and CH_2_Cl_2_ as low polar solvents and *n*-hexane as non-polar solvent. In ethanol and acetonitrile as medium polar solvents (Table 1, entries 11 and 12) and water as high polar solvent, the reaction can be performed, but the yields of 6a were lower than that under solvent-free conditions (Table 1, entry 3). Also, elevating the temperature did not improve the yields of 6a (Table 1, entry 6).

**Table 1:** The effect of solvent, temperature and catalyst loading for the synthesis of 6a^a^

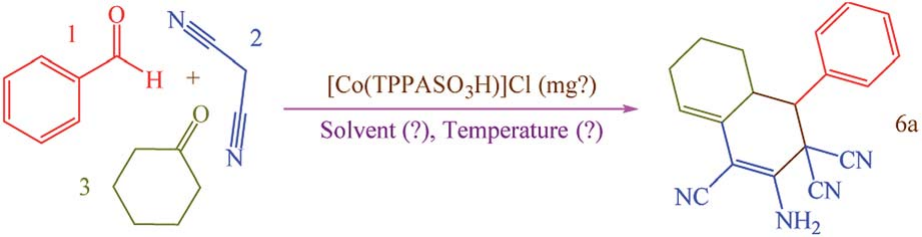					
Entry	Solvent	Catalyst loading (mg)	Temperature (°C)	Time (min)	Yield^b^ (%)
1	—	—	90	180	—
2	—	0.5	90	45	63
3	—	1	90	10	95
4	—	1.5	90	10	88
5	—	2	90	15	88
6	—	1	110	10	90
7	—	1	75	30	73
8	—	1	50	40	60
9	—	1	25	90	—
10	H_2_O	1	Reflux	10	85
11	CH_3_OH	1	Reflux	20	69
12	CH_3_CN	1	Reflux	30	55
13	CH_2_Cl_2_	1	Reflux	120	—
14	EtOAc	1	Reflux	25	35
15	*n*-Hexane	1	Reflux	120	—
16	CHCl_3_	1	Reflux	120	—

a Reaction conditions: benzaldehyde (1 mmol; 0.106 g; 0.102 mL), malononitrile (2 mmol; 0.132 g), cyclohexanone (1 mmol; 0.098 g; 0.104 mL).

b Isolated yield.

Having confirmed the best reaction conditions, the overview of current approach was investigated with a range of aldehydes and ketones (Table 2). It is obvious that aldehydes with electron withdrawing groups usually gave the products 6–9 in shorter reaction time and higher yields in comparison with electron-releasing groups.

**Table 2:** Synthesis of 6–9 in the presence of [Co(TPPASO_3_H)]Cl (1 mg) under solvent-free conditions at 90 °C^a^^,^^b^^,^^c^

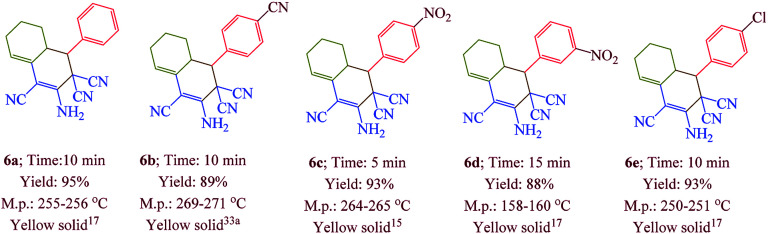
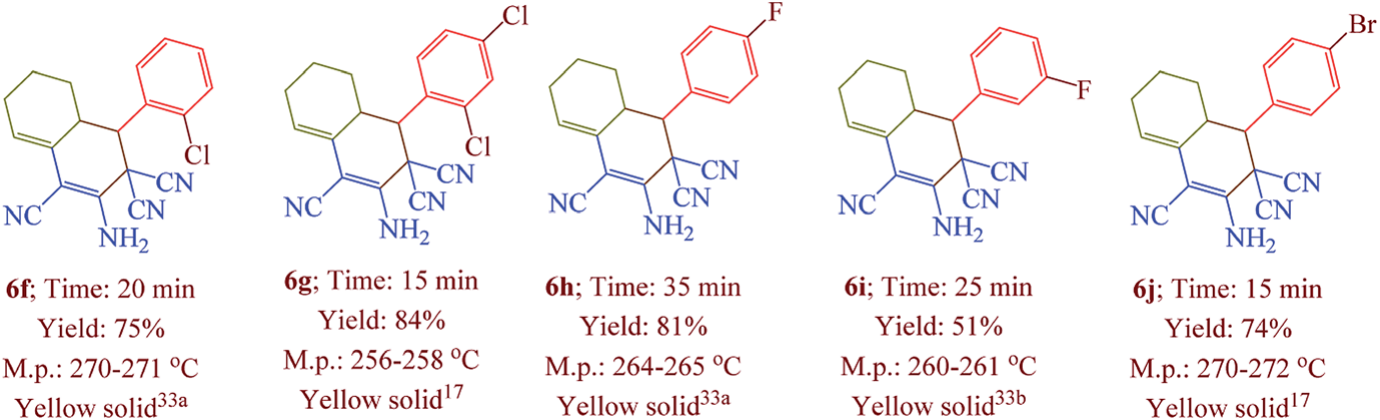
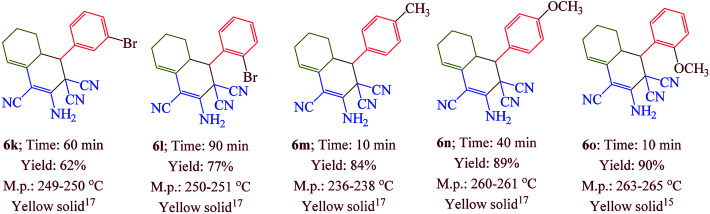
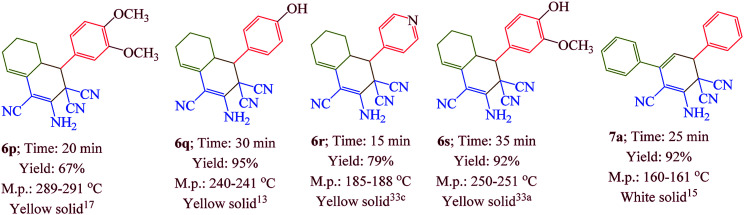
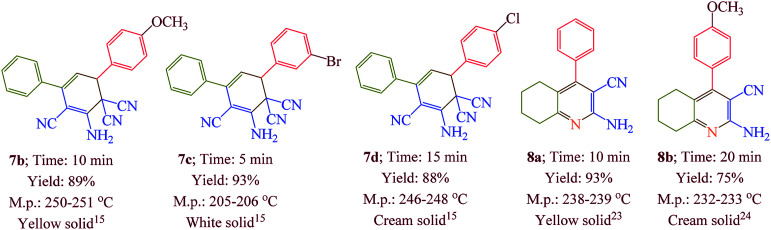
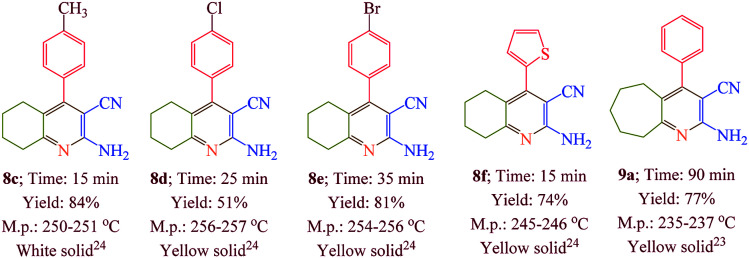

a Reaction conditions: aldehyde (1 mmol), malononitrile (2 mmol; 0.132 g), cyclohexanone (for the synthesis of 6) (1 mmol; 0.098 g; 0.104 mL) or acetophenone (for the synthesis of 7) (1 mmol; 0.120 g; 0.117 mL).

b Reaction conditions: aldehyde (1 mmol), malononitrile (1 mmol; 0.066 g), cyclohexanone (for the synthesis of 8) (1 mmol; 0.098 g; 0.104 mL) or cycloheptanone (for the synthesis of 9) (1 mmol; 0.112 g; 0.118 mL).

c Isolated yield.

**Fig. 7:**
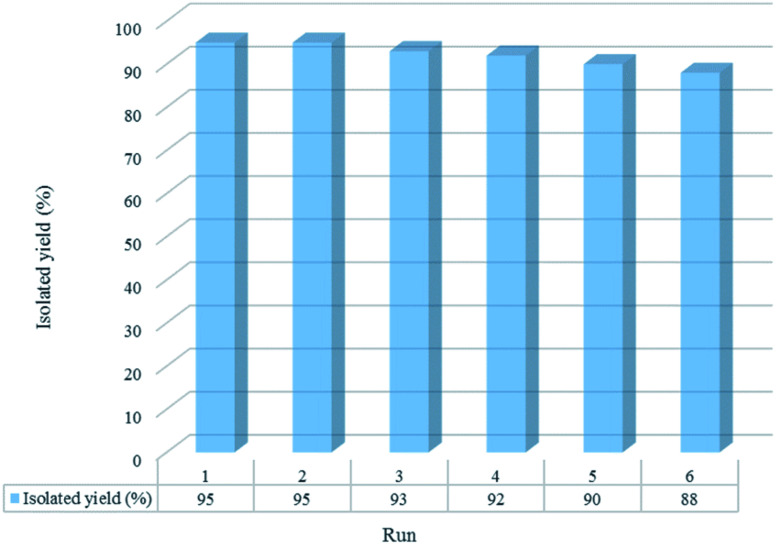
Recyclability study of [Co(TPPASO_3_H)]Cl in the course of synthesis of 6a after 10 min.

**Fig. 8:**
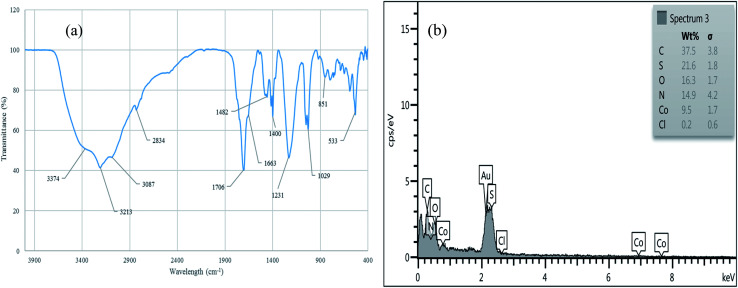
FT-IR (a) and EDX (b) of [Co(TPPASO_3_H)]Cl after six run.

The reusability of [Co(TPPASO_3_H)]Cl was studied in a described model reaction. At the end of reaction, the catalyst was recovered and could be reused six times without any significant decrease in the yield of 6a. After each run, the catalyst was filtered, carefully washed with ethanol (6a is soluble in ethanol but the catalyst is insoluble) and lastly dried for reused in the next run without further purification (Fig. 7). FT-IR and EDX of [Co(TPPASO_3_H)]Cl after six run, confirmed the stability of its structure during the recycling process (Fig. 8). The reaction was scaled up to 10 mmol of benzaldehyde, cyclohexanone and malononitrile for the synthesis of 6a in the presence of 10 mg of [Co(TPPASO_3_H)]Cl under solvent-free condition at 90 °C. The isolated yield of the reaction was 95% after 10 minutes and 88% after the sixth run.

**Scheme 5:**
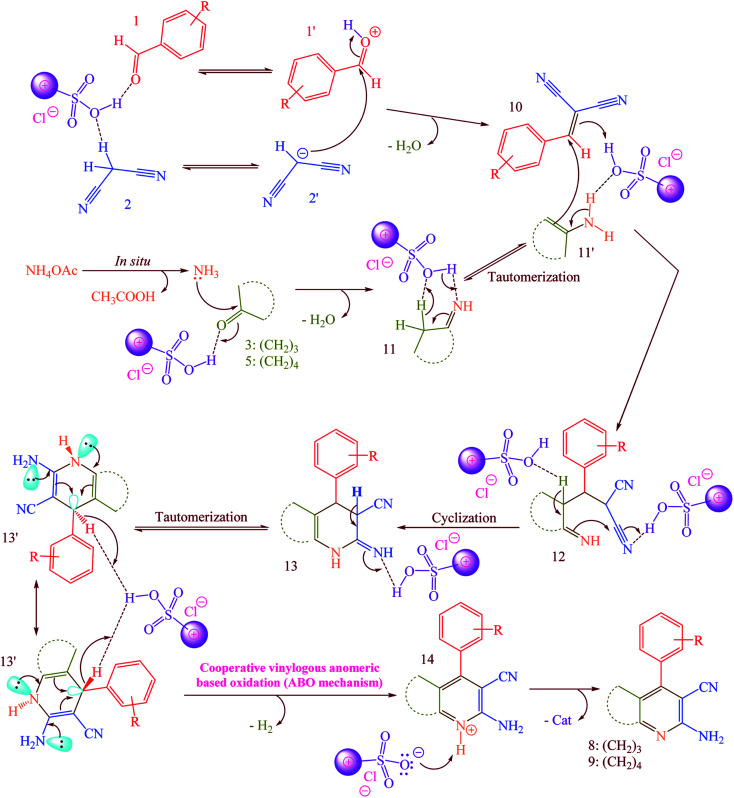
Possible mechanism for the synthesis of 2-amino-3-cyanopyridines (8 and 9) *via* a cooperative vinylogous anomeric based oxidation (ABO) using [Co(TPPASO_3_H)]Cl as a catalyst.

According to the previously proposed mechanism for the synthesis of products 8 or 9, reaction were proceeded by aerobic auto oxidation of 13′ to products 8 or 9 (Scheme 5).^23–25^ In contrast to our expected products 13′, we observed that 13′ was converted to 2-amino-3-cyanopyridines (8 and 9) *via* a hydride transfer which was named anomeric based oxidation (ABO) as well as Cannizzaro reaction (Scheme S1†), H_2_ releasing from tricyclic orthoamide (Scheme S2†) and *etc.*^34–38^ For this reason, reaction was performed without any molecular oxygen under nitrogen and argon atmospheres. It was known that, the reaction proceeded under these conditions as well as normal reaction conditions presence of oxygen. According to this evidence, conversion of 13′ to products 8 or 9 might be occurred by unexpected hydride transfer and releasing of molecular hydrogen (H_2_). Electron donation from the nitrogen lone pairs into the anti-bonding of C–H (σ*_C–H_ orbital) leads to the C–H bond weakened which it can be broken using reaction with a proton to provide molecular hydrogen.

A possible mechanism for the synthesis of 8 or 9 is described in Scheme 5.^23–25^ Firstly, [Co(TPPASO_3_H)]Cl activates the aldehyde 1 and malononitrile 2 to provide intermediate 1′ and 2′, respectively. The Knoevenagel condensation between 1′ and 2′ was happened to produce the arylidenemalononitrile 10. Then, the reaction between ammonium acetate and 3 or 5 was occurred to form the intermediate 11. In the next step, 11 tautomerized to 11′. The reaction between arylidenemalononitrile 10 and intermediate 11′ leads to the intermediate 12 which was cyclized to 13. Then, 13 tautomerized to 13′ as an appropriate structure for supporting by cooperative vinylogous anomeric based oxidation. Finally, driving force of aromatization which was supported by anomeric effect in 13′ leads to hydride transfer and to afford the 2-amino-3-cyanopyridines 8 or 9.

In this research, we synthesized a novel cobalt tetra-2,3-pyridiniumporphyrazinato with sulfonic acid tag [Co(TPPASO_3_H)]Cl. Then, the catalytic activity of this catalyst was investigated for the synthesis of four target molecules with more than 30 derivatives. The reaction times for the synthesis of these compounds were decreased and the isolated yields of produced compounds were increased. Also, the mechanistic route of compounds 8 and 9 were investigated *via* a cooperative vinylogous anomeric based oxidation. This mechanistic route is reported for the first time. To illustration the significance of the present study in comparison with the reported results in the literature, we summarized some of the results for the synthesis of 6e in Table 3, which shows that [Co(TPPASO_3_H)]Cl can act as appropriate catalyst in respect of the reaction time, temperature and displays varied applicability in terms of yield.

**Table 3:** Comparison of the efficiency of [Co(TPPASO_3_H)]Cl in the synthesis of 6e with other reported approaches^a^

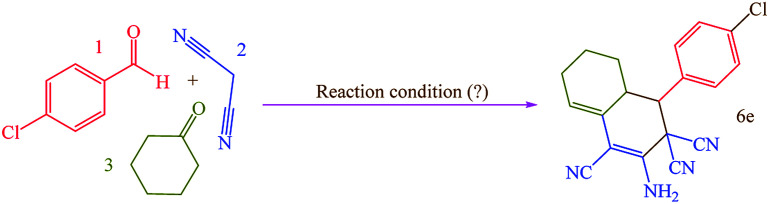				
Entry	Reaction condition	Time (min)	Yield^b^ (%)	Ref.
1	[Co(TPPASO_3_H)]Cl (1 mg), solvent-free, 90 °C	10	93	This work
2	Borax (10 mol%), C_2_H_5_OH, reflux	60	89	14
3	Borax (0.25 mmol), C_2_H_5_OH, reflux	300	90	15
4	Borax (0.1 mL), solvent-free, 80 °C	240	88	15
5	Urea : choline chloride (2 : 1; 0.5 mL), DES, 50 °C	50	86	17
6	Biodegradable IL (20 mol%), C_2_H_5_OH : H_2_O (1 : 1), 60 °C	180	86	33*a*
7	[BPy]BF_4_ (2 mL), 60 °C	240	89	33*b*
8	ZnTiO_3_ (0.2 mmol), H_2_O, rt	180	94	33*c*
9	DABCO (20 mol%), C_2_H_5_OH : H_2_O (70 : 30), reflux	60	94	33*d*
10	[Bmim-G]^+^[Br]^−^ (10 mol%), solvent-free, rt	240	89	33*e*

a Reaction conditions: 4-chlorobenzaldehyde (1 mmol; 0.140 g), malononitrile (2 mmol; 0.132 g), cyclohexanone (1 mmol; 0.098 g; 0.104 mL).

b Isolated yield.

## Conclusion

In summary, we have synthesized cobalt tetra-2,3-pyridiniumporphyrazinato with sulfonic acid tags [Co(TPPASO_3_H)]Cl and its was used for the synthesis of *ortho*-aminocarbonitriles (6), cyclohexa-1,3-dienamines (7) and 2-amino-3-cyanopyridines (8 and 9). This method has major advantages such as suitable yields, wide scope of substrates, avoidance of column chromatography, operational simplicity, simple workup, ready accessible, thermally stable catalyst, minimization of cost and waste generation due to the recycling of the catalyst. The possible mechanism for the synthesis of 2-amino-3-cyanopyridines (8 and 9) was proposed by cooperative vinylogous anomeric based oxidation. More development of this reaction containing investigation on the reaction mechanism and scopes is being followed and will be described in due sequence.

## Conflicts of interest

The authors declare no conflict of interest.

## Supplementary Material

RA-010-D0RA02172E-s001
