# Supplementary material for: Synthesis of cobalt tetra-2,3-pyridiniumporphyrazinato with sulfonic acid tags as an efficient catalyst and its application for the synthesis of bicyclic ortho-aminocarbonitriles, cyclohexa-1,3-dienamines and 2-amino-3-cyanopyridines
Source: RSC Adv. 2020 Jul 24;10(46):27824–34. doi: 10.1039/d0ra02172e (PMC9055596; doi:10.1039/d0ra02172e)

Electronic Supplementary Material (ESI) for RSC Advances.
This journal is © The Royal Society of Chemistry 2020

Supplementary data

Synthesis of cobalt tetra-2,3-pyridiniumporphyrazinato with sulfonic acid tags as an efficient catalyst and its application for the synthesis of bicyclic *ortho*-aminocarbonitriles, cyclohexa-1,3-dienamines and 2-amino-3-cyanopyridines

Mohammad Dashteh,[a] Mohammad Ali Zolfigol,[a]* Ardeshir Khazaei,[a]* Saeed Baghery,[a]* Meysam Yarie,[a] Sajjad Makhdoomi,[b] Maliheh Safaiee[c]*

[a] Department of Organic Chemistry, Faculty of Chemistry, Bu-Ali Sina University, Hamedan 6517838683, Iran

[b] Department of Pharmacology and Toxicology, School of Pharmacy, Hamedan University of Medicinal Science, Hamedan, Iran

[c] Department of Medicinal Plants Production, University of Nahavand, Nahavand, 6593139565, Iran

Fax: +988133493009; E-mail: zolfi@basu.ac.ir and mzolfigol@yahoo.com (M.A. Zolfigol); Khazaei_1326@yahoo.com (A. Khazaei); saadybaghery@yahoo.com (S. Baghery); azalia_s@yahoo.com (M. Safaiee).

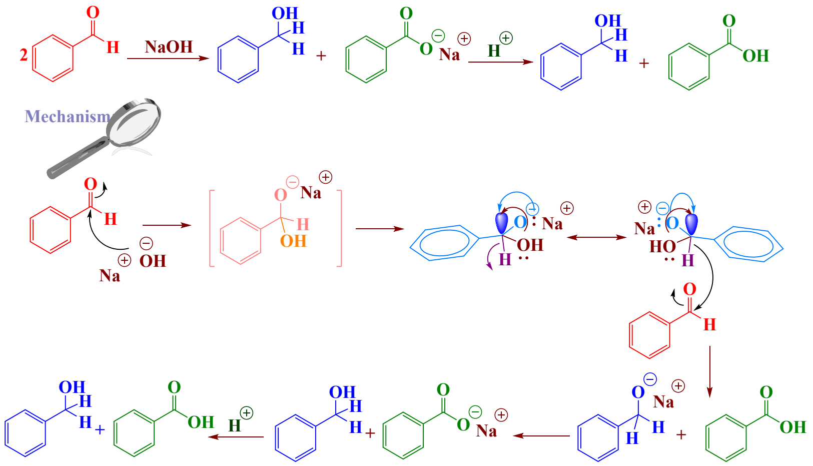

Scheme S1. Suggested mechanism for the in-situ oxidation-reduction Cannizzaro reaction by an uncommon hydride transfer *via* ABO mechanism.

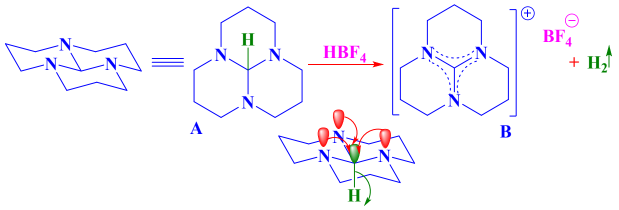

Scheme S2. Unusual hydride transfers from tricyclic orthoamide (A) by ABO mechanism.

General information

The M.p. was determined by Thermo Scientific apparatus. The ^{1}H (300 and 400 MHz) and ^{13}C NMR (75 and 100 MHz) spectra were analyzed by Bruker DRX-400 MHz instrument using TMS as internal standard and DMSO-d_6 as a solvent. FT-IR spectra were analyzed by Perkin-Elmer FT-IR-17259 instrument by KBr disks. Elemental analyses were achieved on a Perkin Elmer 2400 Series II Elemental CHNS analyzer. All the chemicals were purchased from Sigma-Aldrich or Merck and used without further purification. Silica gel plates (Merck) were used for TLC analysis with a mixture of *n*-hexane and ethyl acetate (5:2) as the eluent. Ultraviolet-visible spectra were analyzed by a Perkin-Elmer UV-Vis and DRS Spectrophotometer 35. ICP-MS was performed on an Agilent 7700x apparatus. Elemental compositions were determined with a ZEISS SIGMA VP-500 scanning electron microscope equipped with an X-ray detector Oxford Instrument for microanalysis (SEM-EDX) presenting a 133 eV resolution at 20 kV. TGA and DTA were achieved on a Rheometric Scientific STA 1500 (heating rate of 10 °C min^{-1} up to 600 °C; under a nitrogen atmosphere at 25 °C). XRD was achieved on an analytical X' Pert Pro apparatus with Cu Kα (λ = 0.154 nm) radiation. Solvents were dried, distilled and stored over molecular sieves. Centrifugation method was carried out in an Anke TDL80-2B. FE-SEM images were performed by a Zigma. TEM images were carried out by a Zeiss-EM10C-100 KV.

Selected spectral data analysis for compounds

2-Amino-4a,5,6,7-tetrahydro-4-phenylnaphthalene-1,3,3(4H)-tricarbonitrile (6a)**:** M.p.: 255-256 °C; IR (KBr) *υ*: 3480, 3424, 3366, 3219, 3153, 2205, 1675, 1625, 1539, 1324, 776 cm^{-1}; ^{1}H NMR (300 MHz, DMSO-d_6) (δ, ppm): 0.84-0.90 (m, 1H), 1.45-1.48 (m, 2H), 1.67-1.73 (m, 1H), 2.02-2.08 (m, 1H), 2.17-2.21 (m, 1H), 2.73-2.89 (m, 1H), 3.53 (d, J = 12.0 Hz, 1H), 5.73 (s, 1H), 7.35 (s, 2H), 7.43-7.59 (m, 5H); ^{13}C NMR (75 MHz, DMSO-d_6) (δ, ppm): 135.1, 129.4, 129.3, 129.0, 120.8, 116.6, 113.0, 112.8, 82.0, 51.0, 43.3, 34.3, 27.4, 25.3, 21.4.

2-Amino-4-(4-nitrophenyl)-4a,5,6,7-tetrahydronaphthalene-1,3,3(4H)-tricarbonitrile (6c)**:** Yellow solid; Yield: 93%; M.p.: 264-265 °C; IR (KBr) *υ* (cm^{-1}): 3424.72, 3339.7, 3058.4, 2191.41, 1666.68, 1631.6, 1596.91, 1525.48, 1348.66, 736.35; ^{1}H NMR (400 MHz, DMSO-d_6) (δ, ppm): 0.81-0.88 (m, 1H, –CH_2), 1.43-1.52 (m, 2H, –CH_2), 1.66-1.69 (m, 1H, –CH_2), 2.04-2.05 (m, 2H, –CH_2), 2.15-2.71 (m, 1H, –CH_2), 3.38 (d, J = 12.0 Hz, 1H, –CH), 5.70 (s, 1H, =CH), 6.71-6.86 (dd, 2H, J = 8.0 Hz), 7.19-7.21 (d, 1H, J = 8.0 Hz), 7.33 (s. 2H, –NH_2), 7.35-7.38 (d, 1H, J =

8.0 Hz, Ar-H) ; ^{13}C NMR (100 MHz, DMSO-d_6): δ 157.7, 143.6, 129.0, 124.5, 120.0, 116.1, 114.9, 112.6, 112.5, 81.4, 50.0, 43.2, 33.8, 26.9, 24.8, 21.0.

***2-Amino-4-(4-chlorophenyl)-4a,5,6,7-tetrahydronaphthalene-1,3,3(4H)-tricarbonitrile (6e)*:** M.p.: 250-251 °C; IR (KBr) ν: 3434, 3360, 3308, 3168, 3129, 2185, 1659, 1633, 1522, 1482, 1371, 1153, 762 cm^{-1}; ^{1}H NMR (400 MHz, DMSO-d_6) (δ, ppm): 0.80-0.89 (m, 1H), 1.43-1.46 (m, 2H), 1.63-1.66 (m, 1H), 2.05-2.20 (m, 2H), 2.72-2.79 (m, 1H), 3.60 (d, J = 12.4 Hz, 1H), 5.72 (s, 1H), 7.26 (d, J = 8.0 Hz, 1H), 7.28-7.37 (m, 3H), 7.48-7.64 (m, 2H); ^{13}C NMR (100 MHz, DMSO-d_6) (δ, ppm): 143.3, 134.7, 134.4, 134.2, 129.5, 129.3, 129.2, 129.0, 128.0, 121.0, 116.7, 112.9, 112.8, 82.2, 50.2, 43.2, 34.3, 27.5, 25.4, 21.5.

***2-Amino-4-(2-chlorophenyl)-4a,5,6,7-tetrahydronaphthalene-1,3,3(4H)-tricarbonitrile (6f)*:** M.p.: 270-271 °C; IR (KBr) ν: 3448, 3358, 2949, 2920, 2217, 1633, 1619, 1595, 1270, 1042, 814, 777, 749, 702 cm^{-1}; ^{1}H NMR (400 MHz, DMSO-d_6) (δ, ppm): 0.78-0.87 (m, 1H), 1.36-1.46 (m, 2H), 1.66-1.69 (m, 1H), 2.04-2.21 (m, 2H), 2.86-2.89 (m, 1H), 3.89 (d, J = 12.0 Hz, 1H), 5.77 (s, 1H), 7.42 (s, 2H), 7.48-7.54 (m, 2H), 7.63 (d, J = 8.0 Hz, 1H), 7.77 (d, J = 8.0 Hz, 1H); ^{13}C NMR (100 MHz, DMSO-d_6) (δ, ppm): 143.4, 135.1, 131.8, 130.6, 130.1, 129.1, 128.4, 127.8, 120.9, 115.9, 112.2, 111.5, 81.5, 46.5, 41.6, 34.5, 26.7, 24.7, 20.8.

***2-Amino-4-(2,4-dichlorophenyl)-4a,5,6,7-tetrahydronaphthalene-1,3,3(4H)-tricarbonitrile (6g)*:** Pale Yellow solid; Yield: 92%; M.p.: 260 °C; IR (KBr) *υ* (cm^{-1}): 3411.5, 3334, 3202, 3069.9, 2190.42, 1661.6, 1651.78, 1521.16, 1467.97, 1398.99, 994.07; ^{1}H NMR (400 MHz, DMSO-d_6) (δ, ppm): 0.83-0.95 (m, 1H, –CH_2), 1.52-1.56 (m, 2H, –CH_2), 1.69-1.71 (m, 1H, –CH-$_2$), 2.02-2.23 (m, 2H, –CH_2), 2.72-2.87 (m, 1H, –CH_2), 3.42-3.49 (d, J = 12.0 Hz, 1H, –CH), 5.74 (s, 1H, =CH), 6.99 (s, 1H, Ar-H), 7.11-7.16 (dd, 2H, J = 8.0 Hz, Ar-H), 7.36 (s, 2H, –NH_2); ^{13}C NMR (100 MHz, DMSO-d_6): δ 149.8, 149.4, 149.2, 148.5, 144.1, 144.4, 129.4, 127.3, 127.0, 125.1, 120.6, 119.7, 166.8, 113.4, 113.1, 112.9, 112.3, 111.7, 82.0, 56.1, 55.93 51.1, 50.5, 43.7, 43.4, 34.7, 34.4, 27.4, 25.3, 21.5.

***2-Amino-4-(4-boromophenyl)-4a,5,6,7-tetrahydronaphthalene-1,3,3(4H)-tricarbonitrile (6j)*:** Yellow solid; Yield: 74%; M.p.: 270-272 °C; IR (KBr) *υ* (cm^{-1}): 3428.7, 3336.81, 3259.3, 3179, 2205.77, 1665.87, 1634.70, 1591.63, 1526.61, 1405.36, 1248.61, 1198.62, 784.68, 715.35; ^{1}H NMR (400 MHz, DMSO-d_6) (δ, ppm): 0.80-0.90 (m, 1H, –CH_2), 1.44-1.47 (m, 2H, –CH_2), 1.67-1.68(m, 1H, –CH_2), 2.06-2.21 (m, 2H, –CH_2), 2.50-2.80 (m, 1H, –CH_2), 3.61-3.64 (d, J = 12.0 Hz, 1H, –CH), 5.73 (s, 1H, =CH), 7.27-7.31 (dd, 2H, J = 8.0 Hz), 7.33 (s, 2H, –NH_2), 7.49

(d, 1H, J = 8.0 Hz, Ar-H), 7.65 (d, 1H, J = 8.0 Hz, Ar-H); ^{13}C NMR (100 MHz, DMSO-d_6): δ 157.7, 143.7, 130.0, 128.0, 122.1, 120.5, 120.5, 116.0, 112.5, 111.7, 81.4, 55.7, 42.2, 33.8, 26.7, 24.7, 20.8.

2-Amino-4-(2-boromophenyl)-4a,5,6,7-tetrahydronaphthalene-1,3,3(4H)-tricarbonitrile (6l)**:** Yellow solid; Yield: 77%; M.p.: 250-251 °C; IR (KBr) υ (cm^{-1}): 3417,3305, 3141, 2946, 2214, 1647, 1554, 1520, 1400 ; 1H NMR (300 MHz, DMSO-d6) (δ, ppm): 0.82-0.94 (m, 1H), 1.46-1.50 (m, 2H), 1.68-1.73 (m, 1H), 2.08-2.11 (m, 1H), 2.20-2.25 (m, 1H), 2.79-2.86 (m, 1H), 3.63-3.67 (d, J = 12.0 Hz, 1H), 5.76 (s, 1H), 7.32 (s, 2H), 7.40-7.67 (m, 4H); ^{13}C NMR (75 MHz, DMSO-d6) (δ, ppm): 164.4, 161.2, 143.9, 134.9, 131.4, 129.2, 120.9, 116.6, 112.9, 112.8, 82.1, 34.3, 27.4, 25.3, 21.4.

2-Amino-4a,5,6,7-tetrahydro-4-p-tolylnaphthalene-1,3,3(4H)-tricarbonitrile (6m)**:** M.p.: 236-238 °C; IR (KBr) ν: 3478, 3424, 3366, 3158, 2205, 1674, 1624, 1557, 1540, 818, 777 cm^{-1}; ^{1}H NMR (300 MHz, DMSO-d_6) (δ, ppm): 0.79-0.88 (m, 1H), 1.47-1.50 (m, 2H), 1.66-1.68 (m, 1H), 2.00-2.06 (m, 1H), 2.16-2.21 (m, 1H), 2.34 (s, 3H), 2.75-2.80 (m, 1H), 3.48 (d, *J* = 12.0 Hz, 1H), 5.71 (s, 1H), 7.23 (s, 1H), 7.29 (s, 1H), 7.37 (s, 2H), 7.46 (s, 1H); ^{13}C NMR (75 MHz, DMSO-d_6) (δ, ppm): 144.0, 138.7, 132.7, 132.0, 129.7, 129.3, 127.2, 120.7, 116.6, 113.0, 112.8, 82.0, 50.7, 43.4, 34.3, 27.4, 25.3, 21.4, 21.2.

2-Amino-4-(4-methoxyphenyl)-4a,5,6,7-tetrahydronaphthalene-1,3,3(4H)-tricarbonitrile (6n)**:** Yellow solid; Yield: 89%; M.p.: 260-261 °C; IR (KBr) *υ* (cm^{-1}): 3409.01, 3333.09, 3218.48, 2968.08, 2199.66, 1683.85, 1660.55, 1622.00, 1469.13, 1374.02, 1215.83, 994.69, 847.31, 782.78; ^{1}H NMR (400 MHz, DMSO-d_6) (δ, ppm): 0.75-0.82 (m, 1H, –CH_2), 1.48-1.52 (m, 2H, –CH_2), 1.65-1.68 (m, 1H, –CH_2), 2.08-2.16 (m, 2H, –CH_2), 2.73-2.80 (m, 1H, –CH_2), 3.75 (s, 3H, –CH_3), 3.84-3.87 (d, J = 12.0 Hz, 1H, –CH), 5.73 (s, 1H, =CH), 7.08-7.15 (dd, 2H, J = 8.0 Hz, Ar-H), 7.34 (s. 2H, –NH_2), 7.39-7.54 (dd, 2H, J = 8.0 Hz, Ar-H) ; ^{13}C NMR (100 MHz, DMSO-d_6): δ 157.7, 143.7, 130.0, 128.9, 128.8, 128.0, 122.1, 120.6, 120.5, 120.5, 116.0, 112.5, 111.9, 111.7, 81.4, 55.7, 42.2, 33.8, 26.7, 24.7, 20.8.

2-Amino-4a,5,6,7-tetrahydro-4-(3,4-dimethoxyphenyl)naphthalene-1,3,3(4H)-tricarbonitrile (6p)**:** M.p.: 289-291 °C; IR (KBr) ν: 3433, 3335, 2939, 2864, 2212, 1649, 1519, 1264, 1021, 823, 766, 419 cm^{-1}; ^{1}H NMR (500 MHz, DMSO-d_6) (δ, ppm): 0.86-0.88 (m, 1H), 1.50-1.53 (m, 2H), 1.67-1.69 (m, 1H), 1.99-2.20 (m, 2H), 2.73-2.82 (m, 1H), 3.33-3.46 (t, *J* = 12.0 Hz, 1H), 3.73-3.78 (d, 6H), 5.71 (s, 1H), 6.96 (s, 1H), 7.08-7.14 (m, 2H), 7.34 (s, 2H); ^{13}C NMR

(125 MHz, DMSO-d_6): δ 162.2, 143.5, 128.9, 126.9, 124.7, 120.3, 119.2, 116.2, 112.5, 111.9, 111.3, 110.3, 81.6, 55.6, 50.8, 50.4, 43.1, 35.7, 30.7, 26.9, 24.9, 21.0.

2-Amino-4-(4-methoxyphenyl)-5,6,7,8-tetrahydroquinoline-3-carbonitrile (8b)**:** Cream solid; Yield: 75%; M.p : 232-233 °C; IR (KBr) ν: 3420.09, 3340.63, 3254.93, 2961.92, 2935.82, 2830.19, 2210.74, 1650.58, 1601.48, 1454.49, 1392.88, 714.02 cm^{-1}; ^1H NMR (500 MHz, DMSO-d_6) (δ, ppm): 1.62-1.63 (m, 2H, $–CH_2$), 1.75-1.76 (m, 2H, $–CH_2$), 2.18-2.21 (br s, 2H, $–CH_2$), 2.72-2.74 (2H, br s, CH_2), 3.78 (s, 3H, $–CH_3$), 6.73 (s, 2H, $–NH_2$), 7.65-7.67 (d, 2H, Ar-H), 8.36-8.39 (d, 2H, Ar-H) ; ^{13}C NMR (125 MHz, DMSO-d_6): 161.9, 158.3, 152.2, 148.0, 143.6, 130.4, 124.2, 118.3, 116.7, 87.6, 55.5, 33.3, 31.1, 26.1, 22.8, 22.4.

2-Amino-4-(4-chlorophenyl)-5,6,7,8-tetrahydroquinoline-3-carbonitrile (8e)**:** M.p.: 256-257 °C; IR (KBr) ν: 3421.01, 3343.82, 3253.01, 2948.35, 2833.26, 2212.72, 1645.85, 16.02.96, 1094.80 cm^{-1}; ^1H NMR (500 MHz, DMSO-d_6) (δ, ppm): 1.61-1.75 (4H, m, CH_2), 2.18-2.20 (2H, brs, CH_2), 2.72-2.74 (2H, brs, CH_2), 6.72 (2H, s, NH_2), 7.64-7.66 (2H, d, ArH), 8.35-8.37 (2H, d, ArH); ^{13}C NMR (125 MHz, DMSO-d_6): 161.9, 158.3, 152.2, 148.0, 143.6, 130.4, 124.2, 118.3, 116.7, 87.6, 33.3, 26.1, 22.8, 22.4.

2-*Amino-4-(thiophen-2-yl)-5,6,7,8-tetrahydroquinoline-3-carbonitrile (8f)*: Cream solid; Yield: 75%; M.p : 232-233 °C ; IR (KBr) ν: 3422.34, 3337.37, 3251.70, 2948.34, 2828.88, 2213.12, 1647.99, 1600.21, 1211.94, 829 cm^{-1}; ^1H NMR (500 MHz, DMSO-d_6) (δ, ppm): 1.59-1.63 (m, 2H, $–CH_2$), 1.74-1.78 (m, 2H, $–CH_2$), 2.20-2.24 (br s, 2H, $–CH_2$), 2.70-2.75 (2H, br s, CH_2), 6.60 (s, 2H, $–NH_2$), 7.35-7.39 (m, 3H, Ar-H) ; ^{13}C NMR (125 MHz, DMSO-d_6): 161.5, 153.3, 133.1, 130.8, 118.9, 117.0, 116.1, 115.9, 88.5, 33.3, 31.1, 26.2, 22.9, 22.5.

FT-IR, ^{1}H NMR and ^{13}C NMR spectra

Fig. S1. FT-IR spectrum of 2-amino-4a,5,6,7-tetrahydro-4-phenylnaphthalene-1,3,3(4H)-tricarbonitrile (6a)

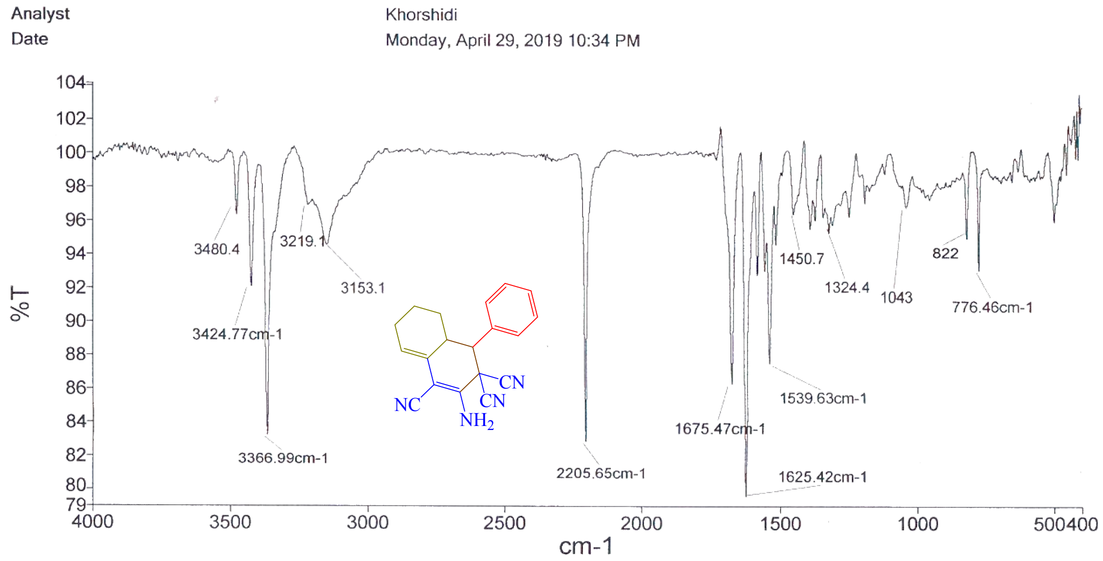

Fig. S2. ^{1}H NMR spectrum of 2-amino-4a,5,6,7-tetrahydro-4-phenylnaphthalene-1,3,3(4H)-tricarbonitrile (6a)

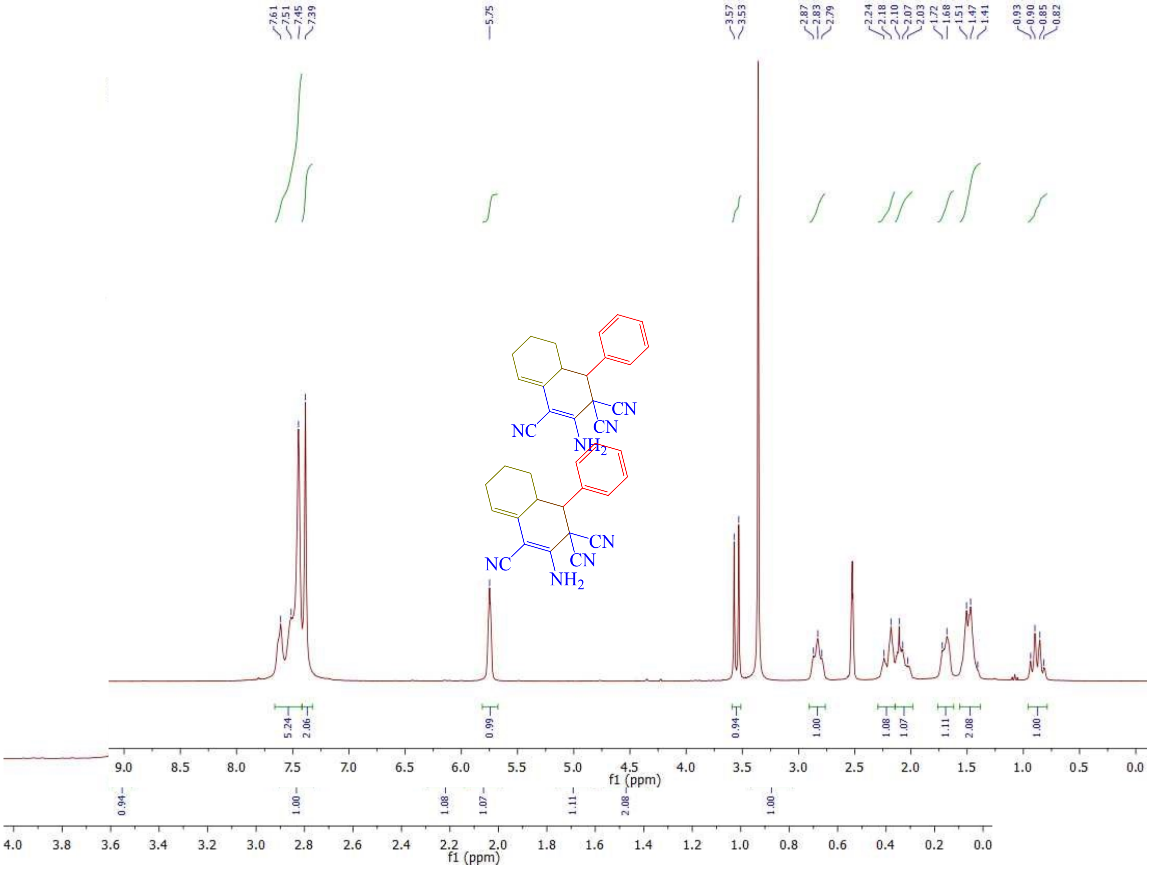

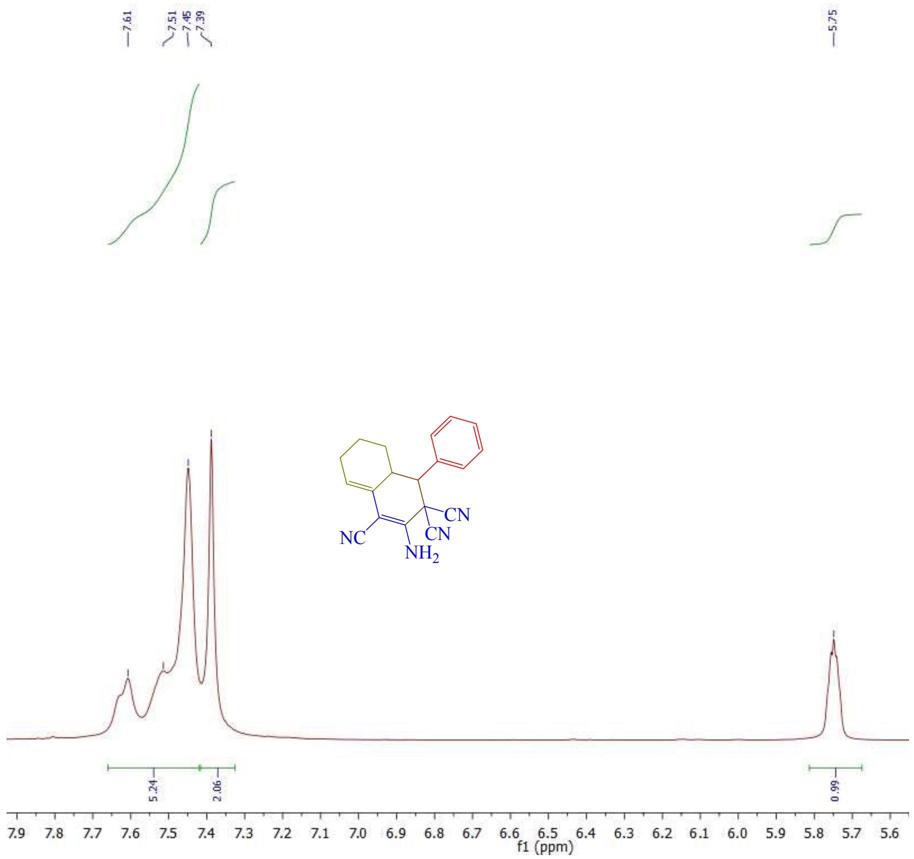

Fig. S3. ^{13}C NMR spectrum of 2-amino-4a,5,6,7-tetrahydro-4-phenylnaphthalene-1,3,3(4H)-tricarbonitrile (6a)

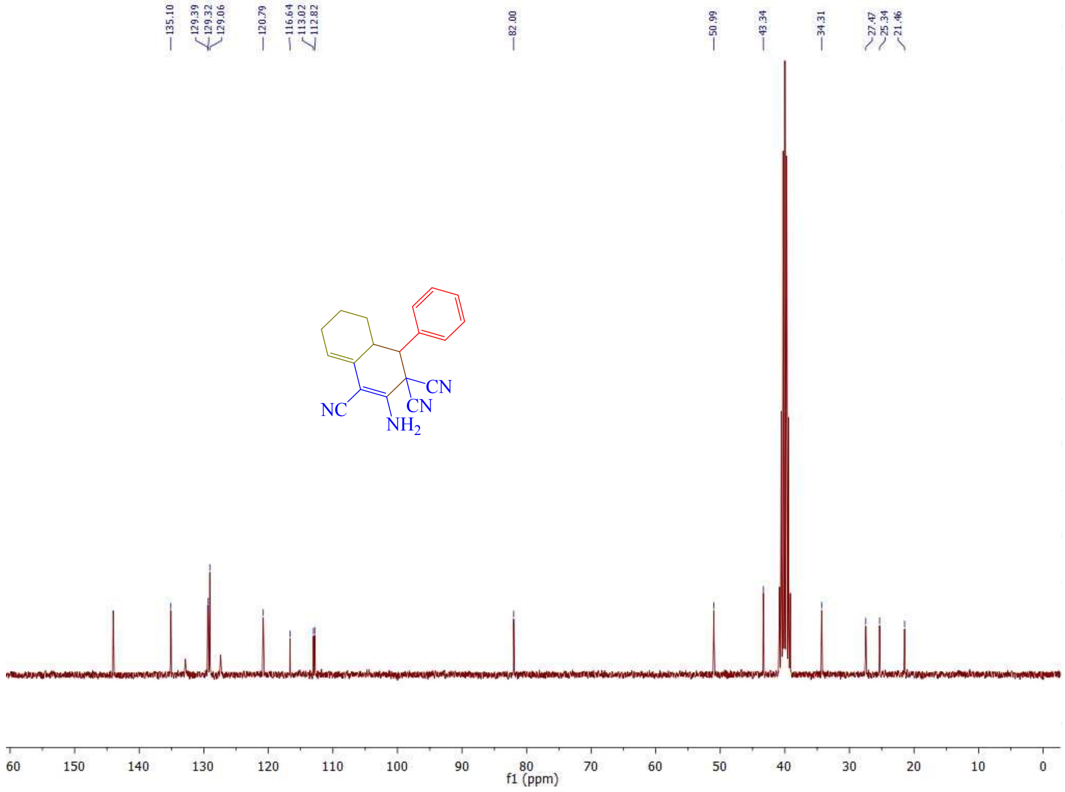

Fig. S4. FT-IR spectrum of 2-amino-4-(4-nitrophenyl)-4a,5,6,7-tetrahydronaphthalene-1,3,3(4*H*)-tricarbonitrile (6c)

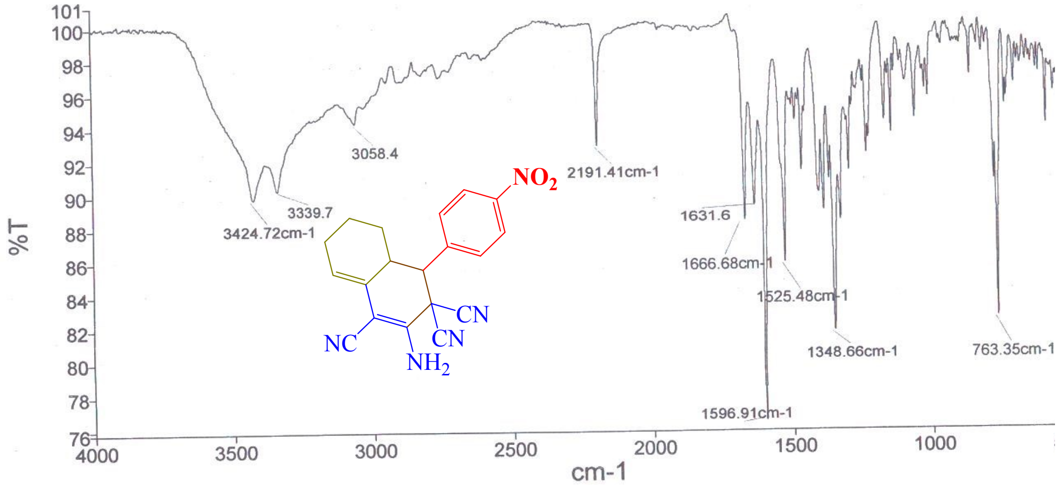

Fig. S5. ^{1}H NMR spectrum of 2-amino-4-(4-nitrophenyl)-4a,5,6,7-tetrahydronaphthalene-1,3,3(4*H*)-tricarbonitrile (6c)

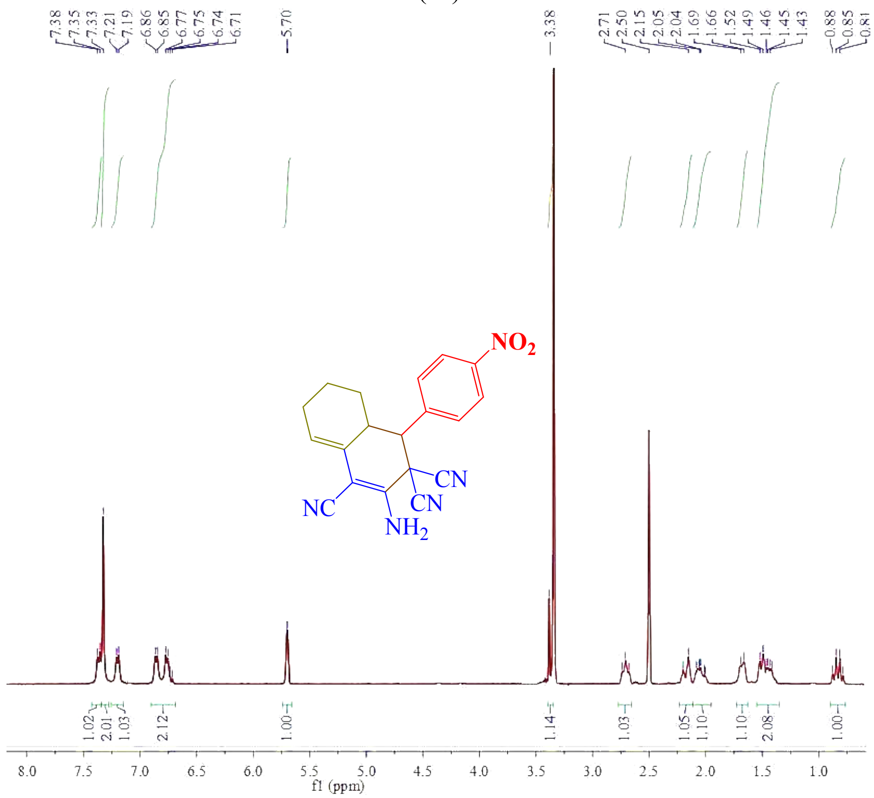

Fig. S6. ^{13}C NMR spectrum of 2-amino-4-(4-nitrophenyl)-4a,5,6,7-tetrahydronaphthalene-1,3,3(4*H*)-tricarbonitrile (6c)

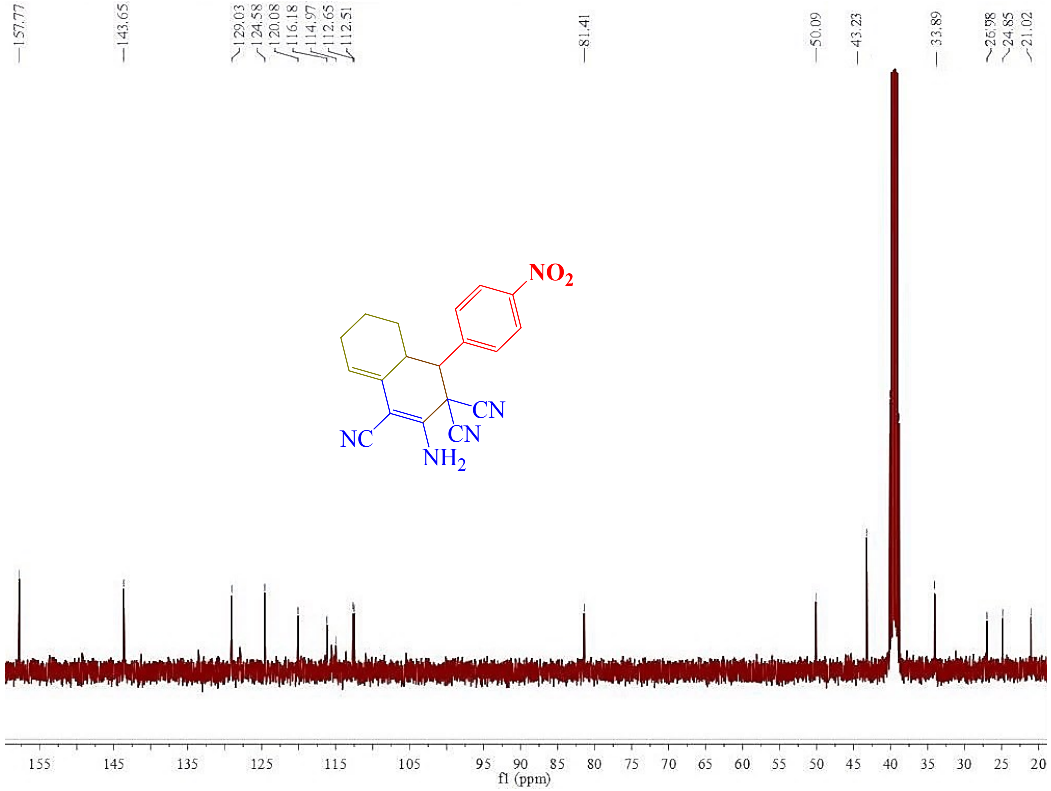

Fig. S7. FT-IR spectrum of 2-amino-4-(4-chlorophenyl)-4a,5,6,7-tetrahydronaphthalene-1,3,3(4*H*)-tricarbonitrile (6e)

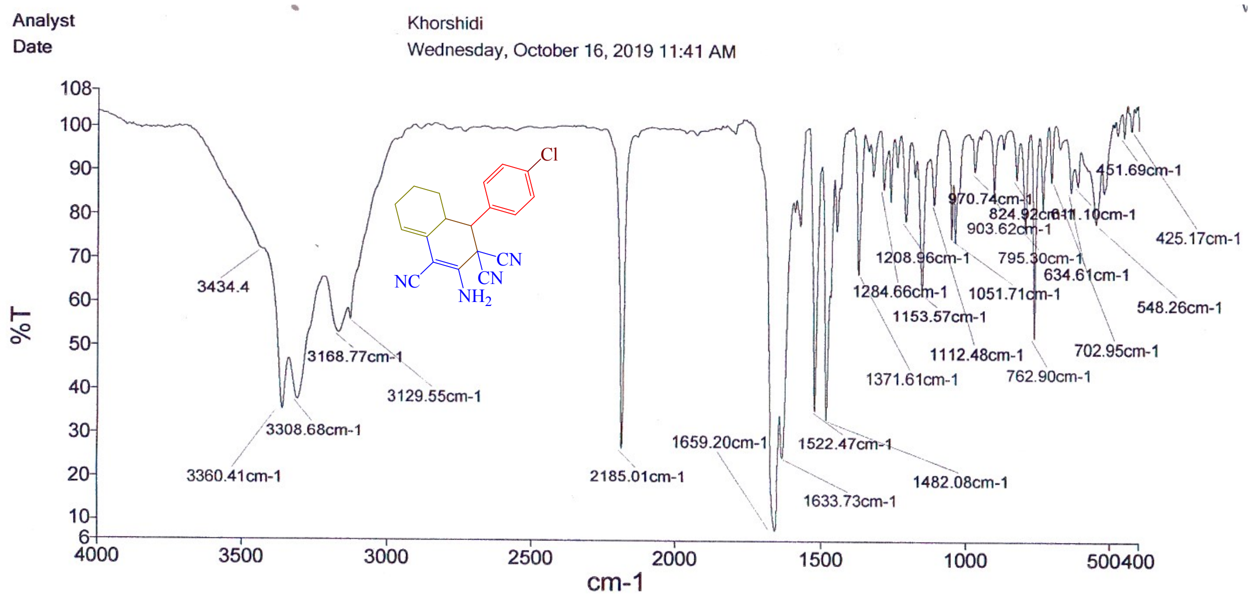

Fig. S8. ^{1}H NMR spectrum of 2-amino-4-(4-chlorophenyl)-4a,5,6,7-tetrahydronaphthalene-1,3,3(4*H*)-tricarbonitrile (6e)

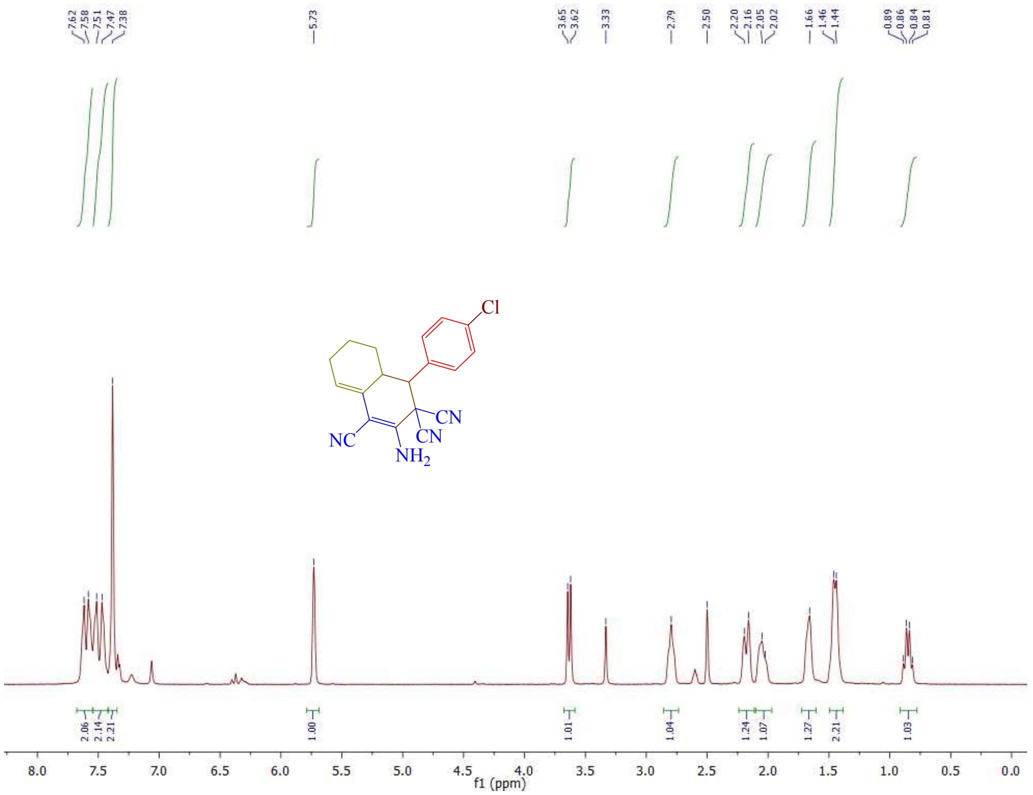
Cl
CN
CN
NC
NH2
f1 (ppm)

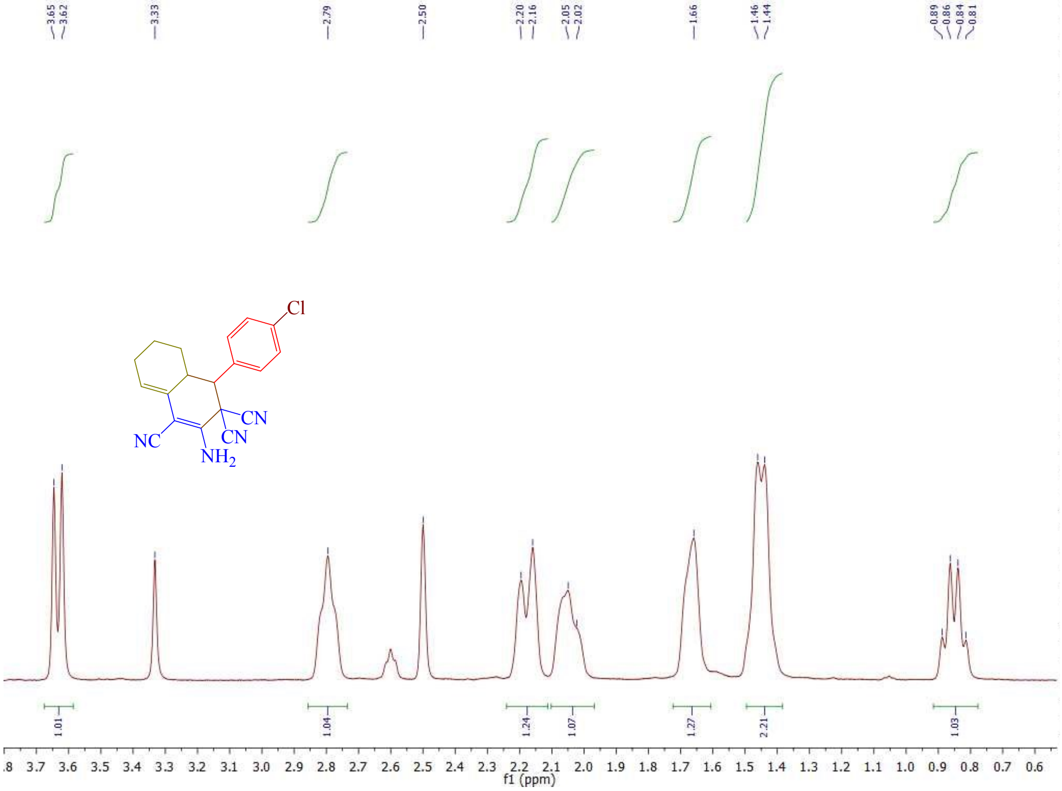
Cl
CN
CN
NC
NH2
f1 (ppm)

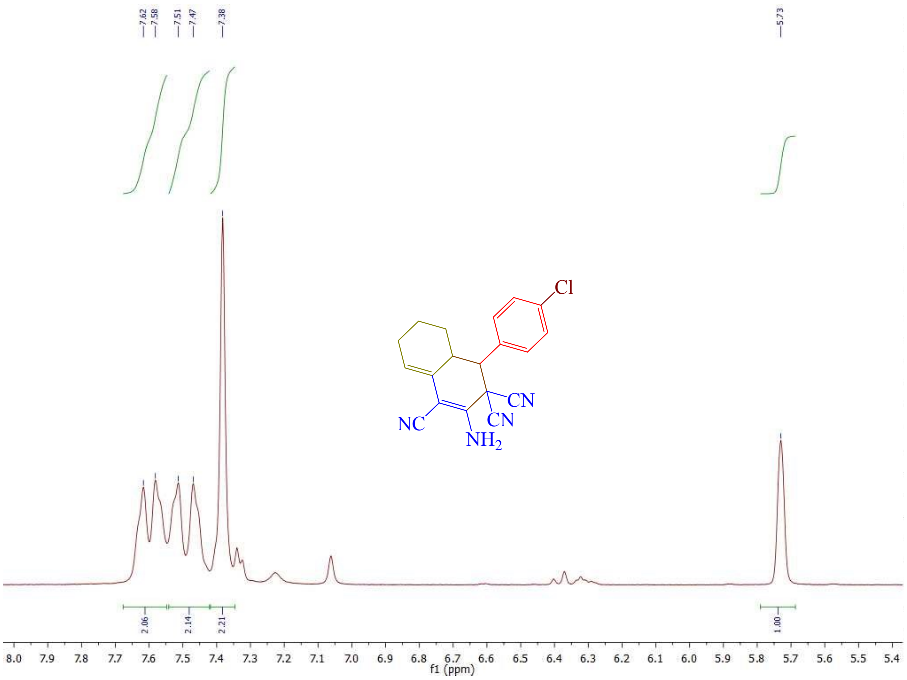

Fig. S9. ^{13}C NMR spectrum of 2-amino-4-(4-chlorophenyl)-4a,5,6,7-tetrahydronaphthalene-1,3,3(4*H*)-tricarbonitrile (6e)

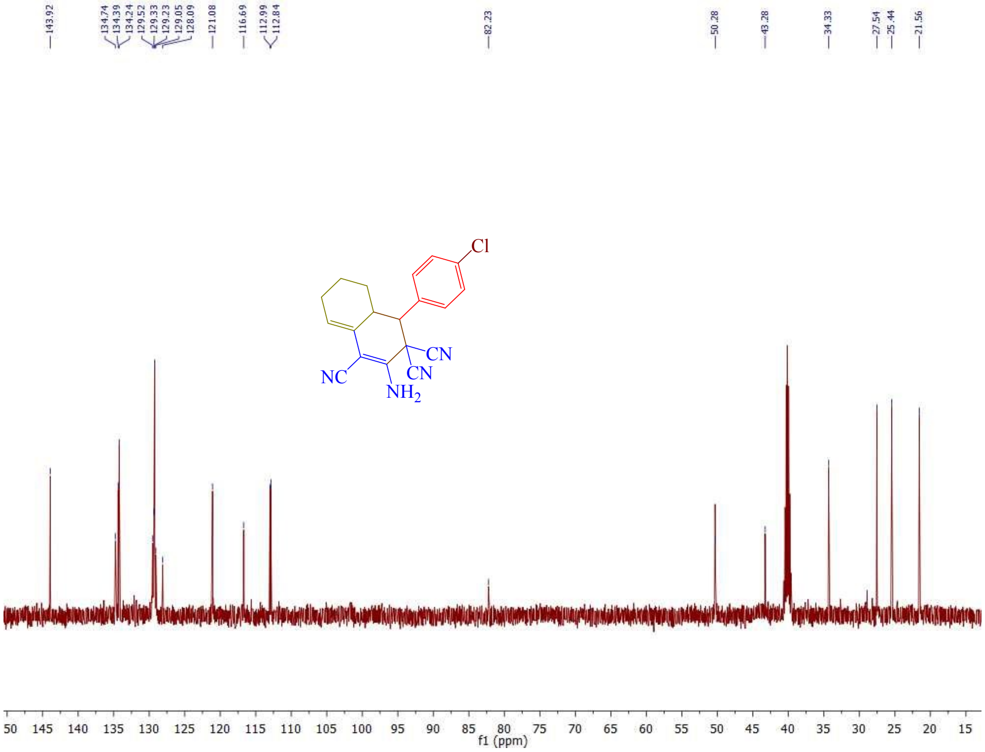

Fig. S10. FT-IR spectrum of 2-amino-4-(2-chlorophenyl)-4a,5,6,7-tetrahydronaphthalene-1,3,3(4*H*)-tricarbonitrile (6f)

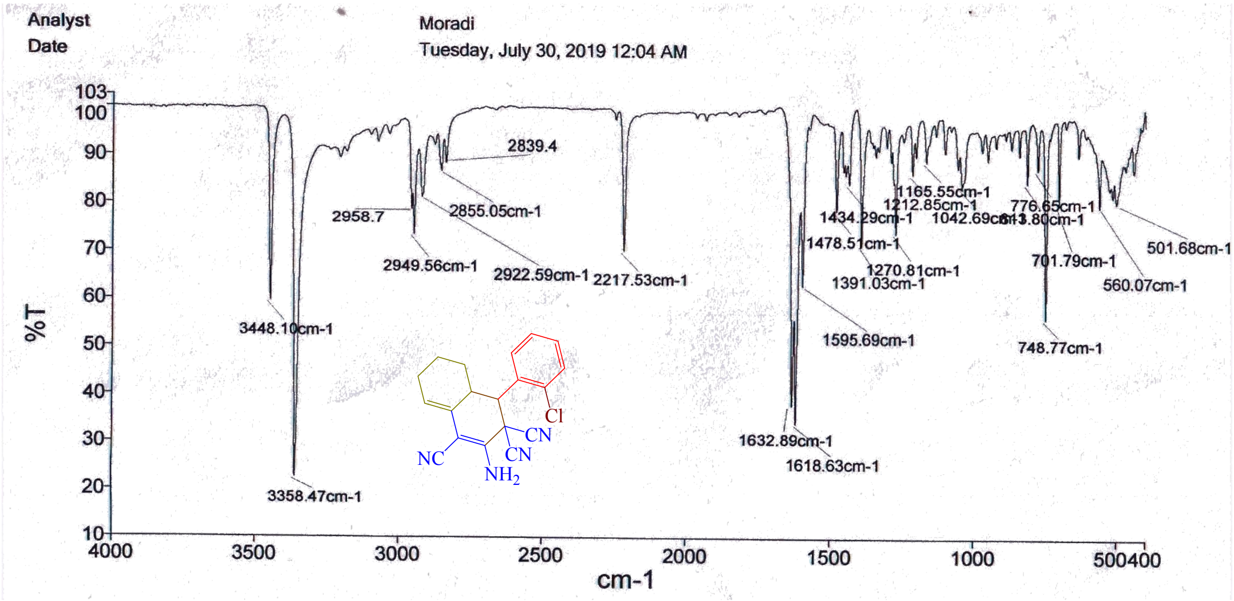

Fig. S11. ^{1}H NMR spectrum of 2-amino-4-(2-chlorophenyl)-4a,5,6,7-tetrahydronaphthalene-1,3,3(4*H*)-tricarbonitrile (6f)

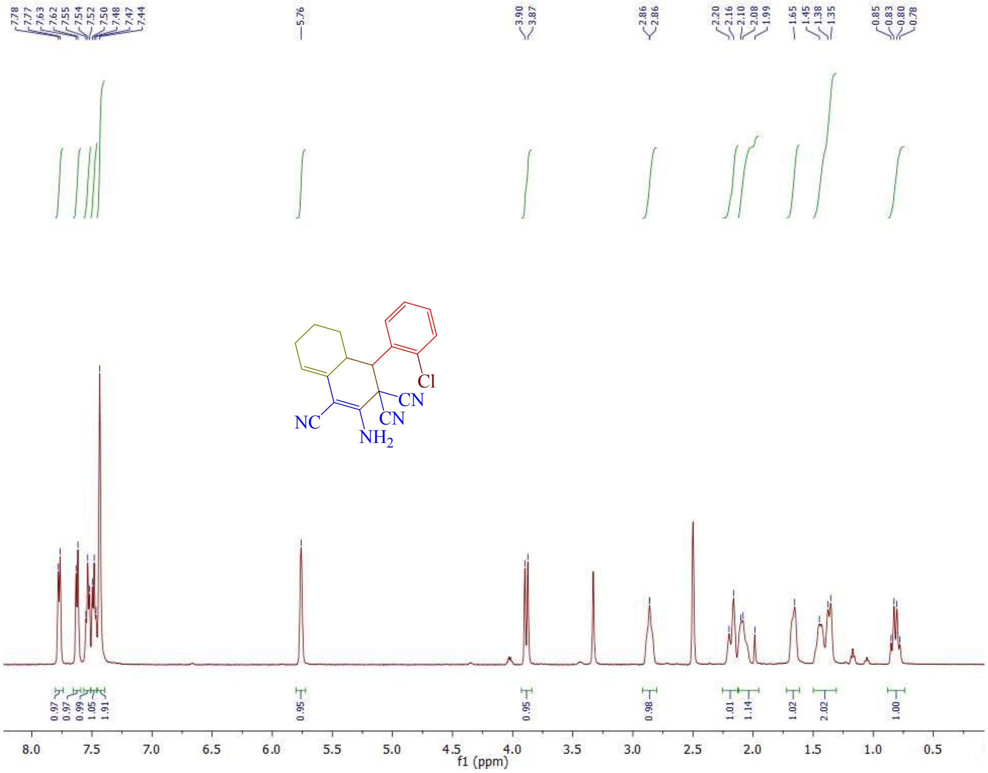

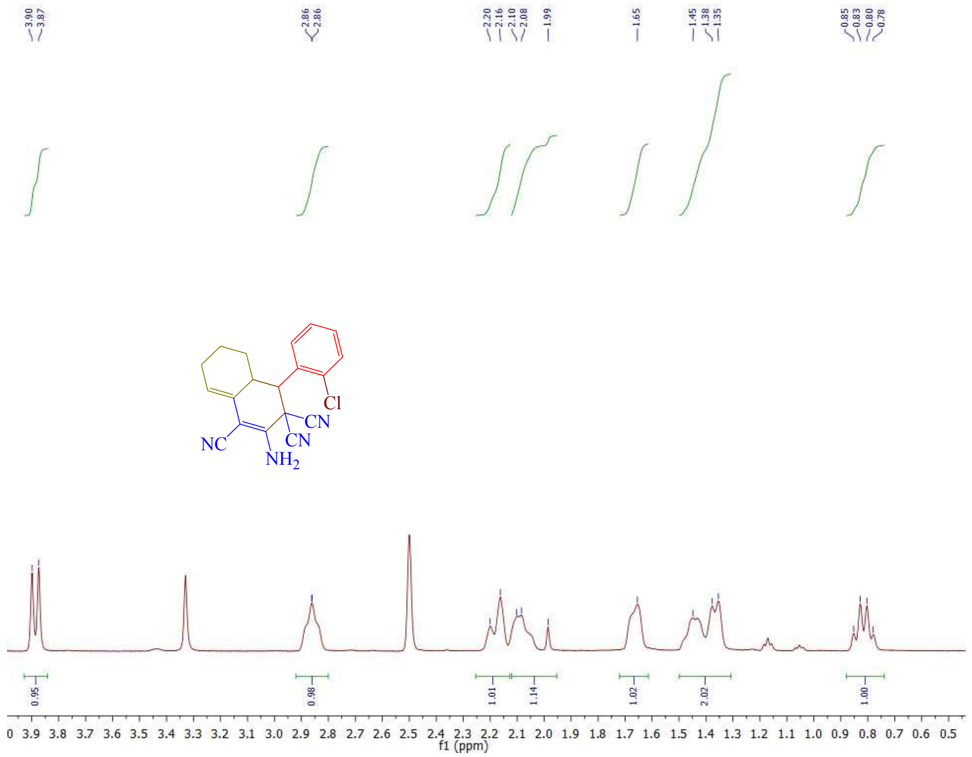
3.90
3.87
2.86
2.86
2.20
2.16
2.10
2.08
1.99
1.65
1.45
1.38
1.35
0.85
0.83
0.80
0.78
Cl
CN
CN
NC
NH2
0.95
0.98
1.01
1.14
1.02
2.02
1.00
f1 (ppm)

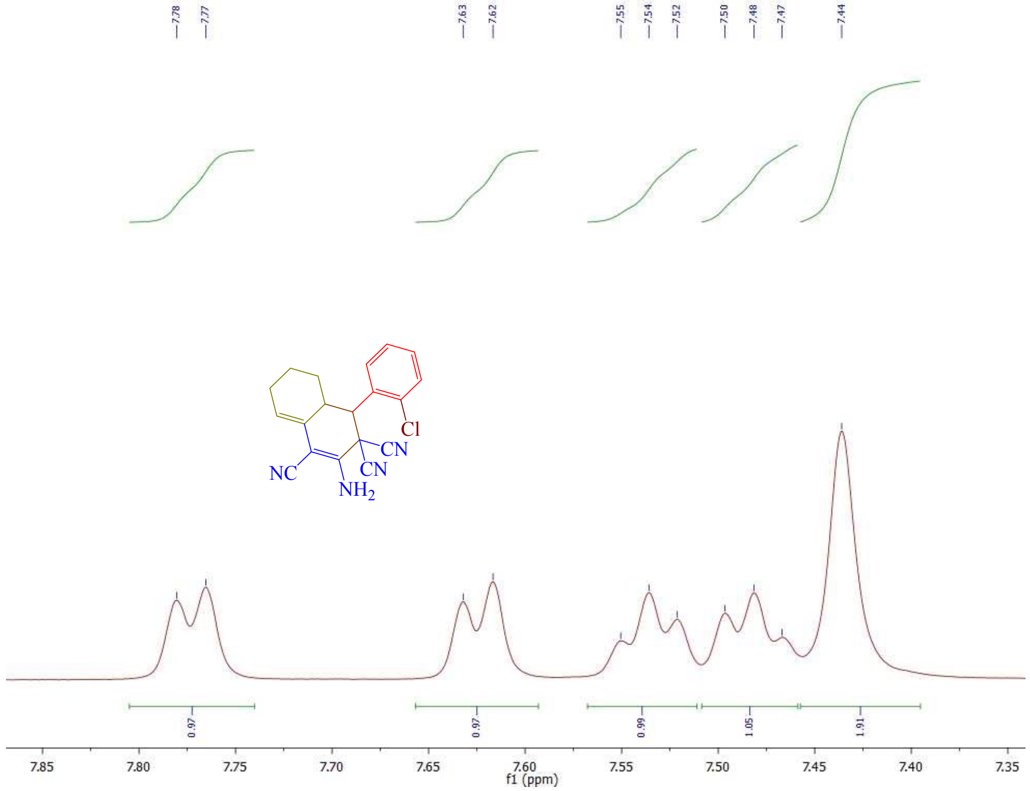
7.78
7.77
7.63
7.62
7.55
7.54
7.52
7.50
7.48
7.47
7.44
Cl
CN
CN
NC
NH2
0.97
0.97
0.99
1.05
1.91
f1 (ppm)

Fig. S12. ^{13}C NMR spectrum of 2-amino-4-(2-chlorophenyl)-4a,5,6,7-tetrahydronaphthalene-1,3,3(4*H*)-tricarbonitrile (6f)

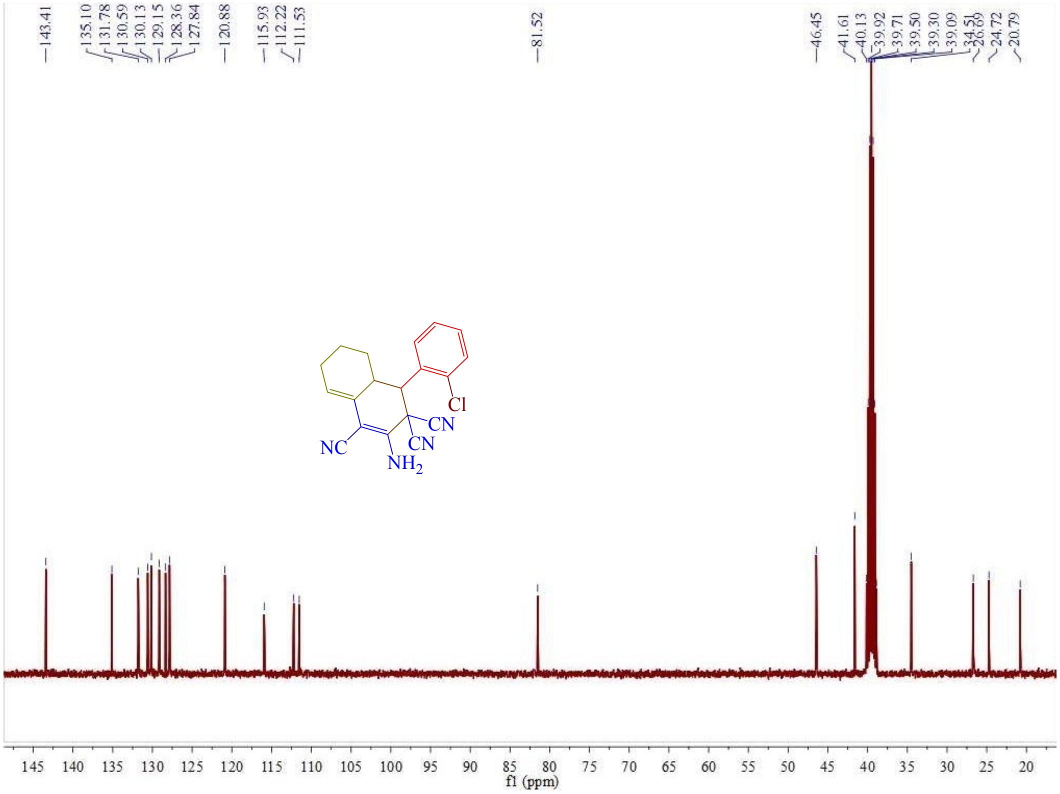

Fig. S13. FT-IR spectrum of 2-amino-4-(2,4-dichlorophenyl)-4a,5,6,7-tetrahydronaphthalene-1,3,3(4*H*)-tricarbonitrile (6g)

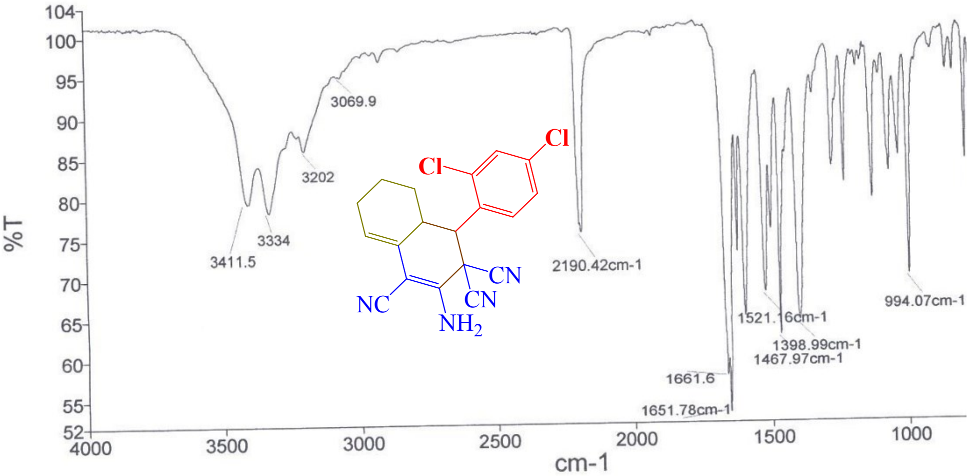

Fig. S14. ^{1}H NMR spectrum of 2-amino-4-(2,4-dichlorophenyl)-4a,5,6,7-tetrahydronaphthalene-1,3,3(4*H*)-tricarbonitrile (6g)

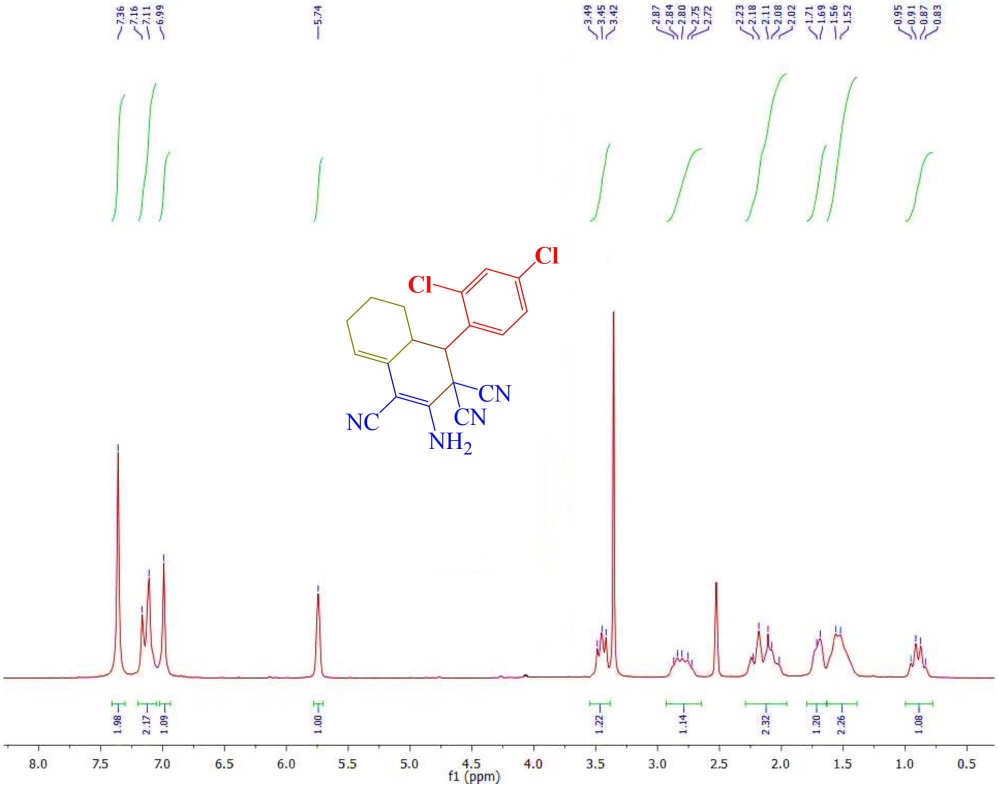

Fig. S15. ^{13}C NMR spectrum of 2-amino-4-(2,4-dichlorophenyl)-4a,5,6,7-tetrahydronaphthalene-1,3,3(4*H*)-tricarbonitrile (6g)

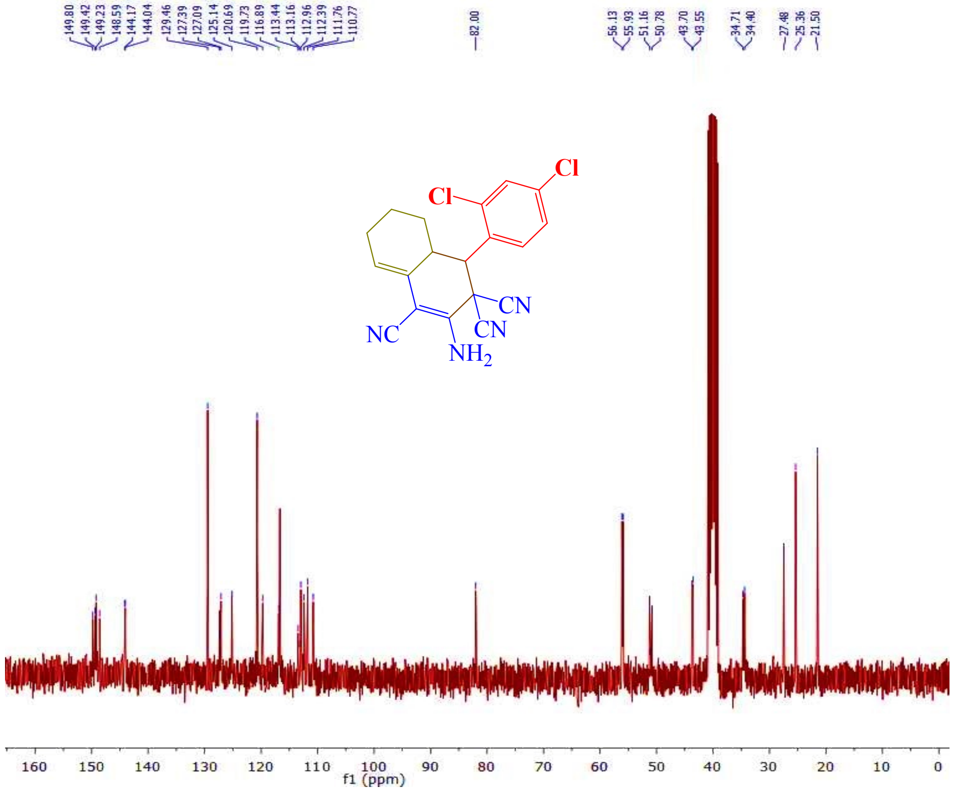

Fig. S16. FT-IR spectrum of 2-amino-4-(4-boromophenyl)-4a,5,6,7-tetrahydronaphthalene-1,3,3(4*H*)-tricarbonitrile (6j)

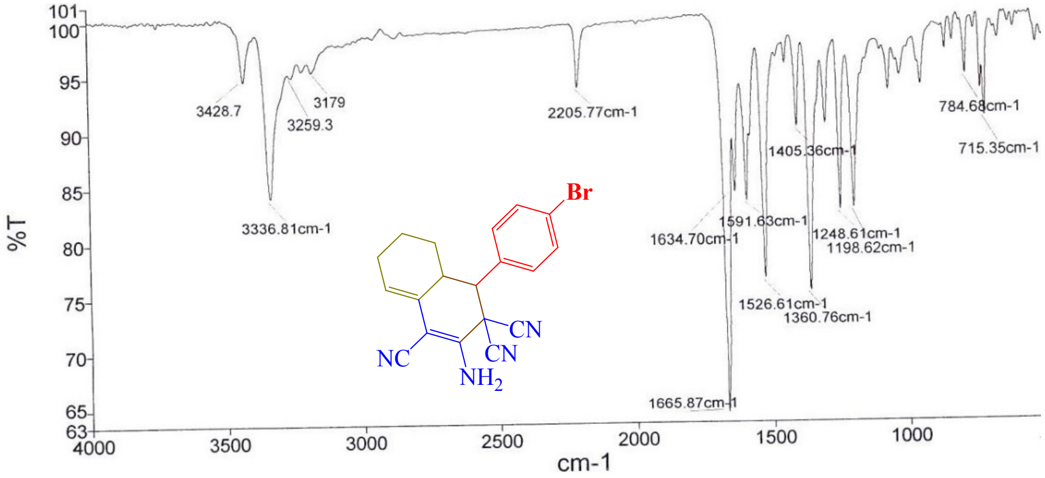

Fig. S17. ^{1}H NMR spectrum of 2-amino-4-(4-boromophenyl)-4a,5,6,7-tetrahydronaphthalene-1,3,3(4*H*)-tricarbonitrile (6j)

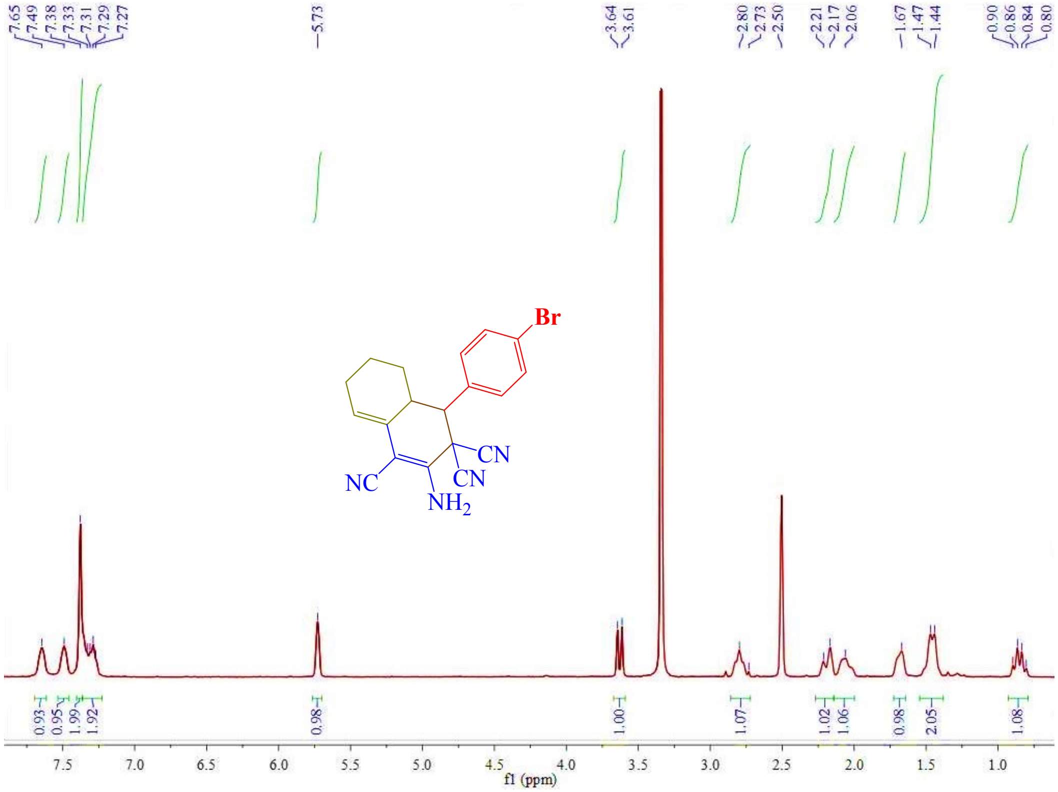

Fig. S18. ^{13}C NMR spectrum of 2-amino-4-(4-boromophenyl)-4a,5,6,7-tetrahydronaphthalene-1,3,3(4*H*)-tricarbonitrile (6j)

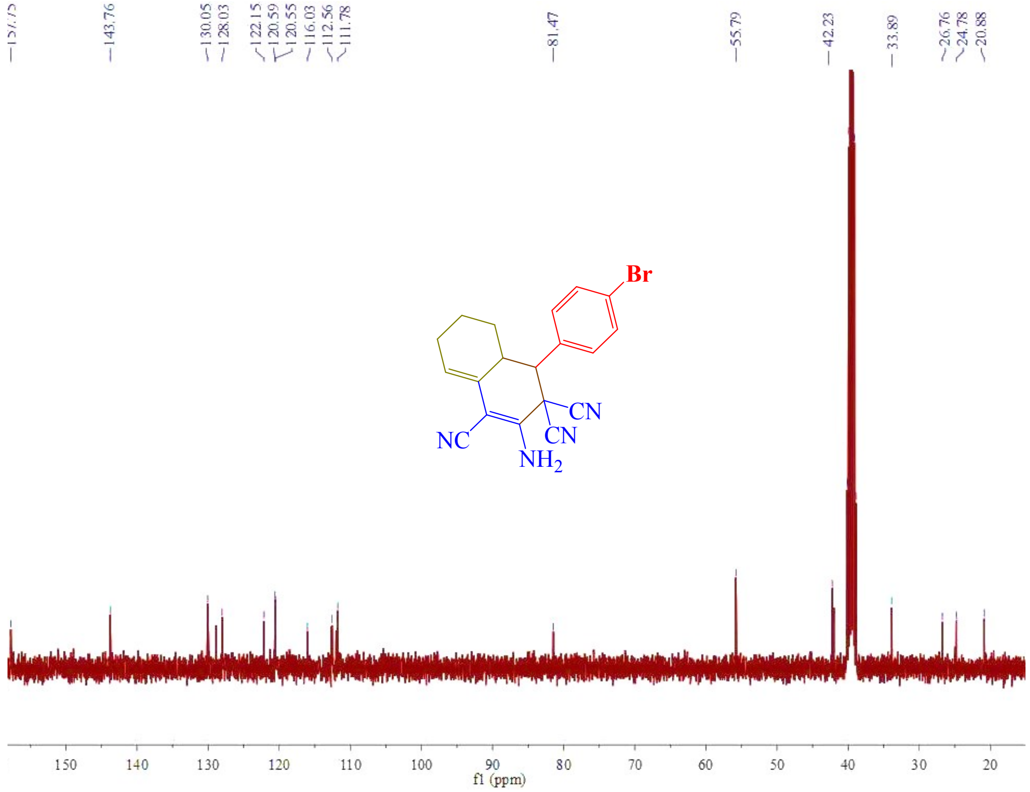

Fig. S19. FT-IR spectrum of 2-amino-4-(2-boromophenyl)-4a,5,6,7-tetrahydronaphthalene-1,3,3(4*H*)-tricarbonitrile (6l)

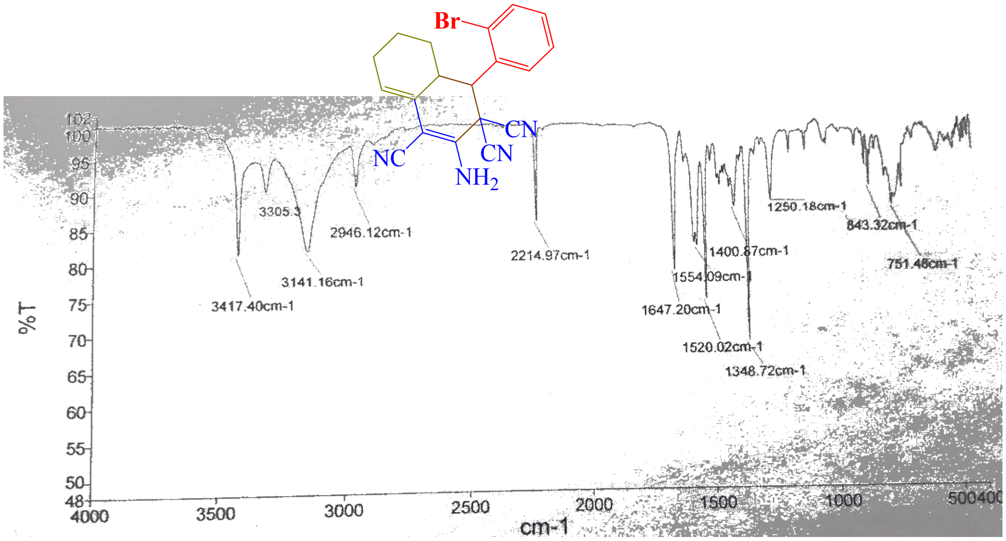

Fig. S20. ^{1}H NMR spectrum of 2-amino-4-(2-boromophenyl)-4a,5,6,7-tetrahydronaphthalene-1,3,3(4*H*)-tricarbonitrile (6l)

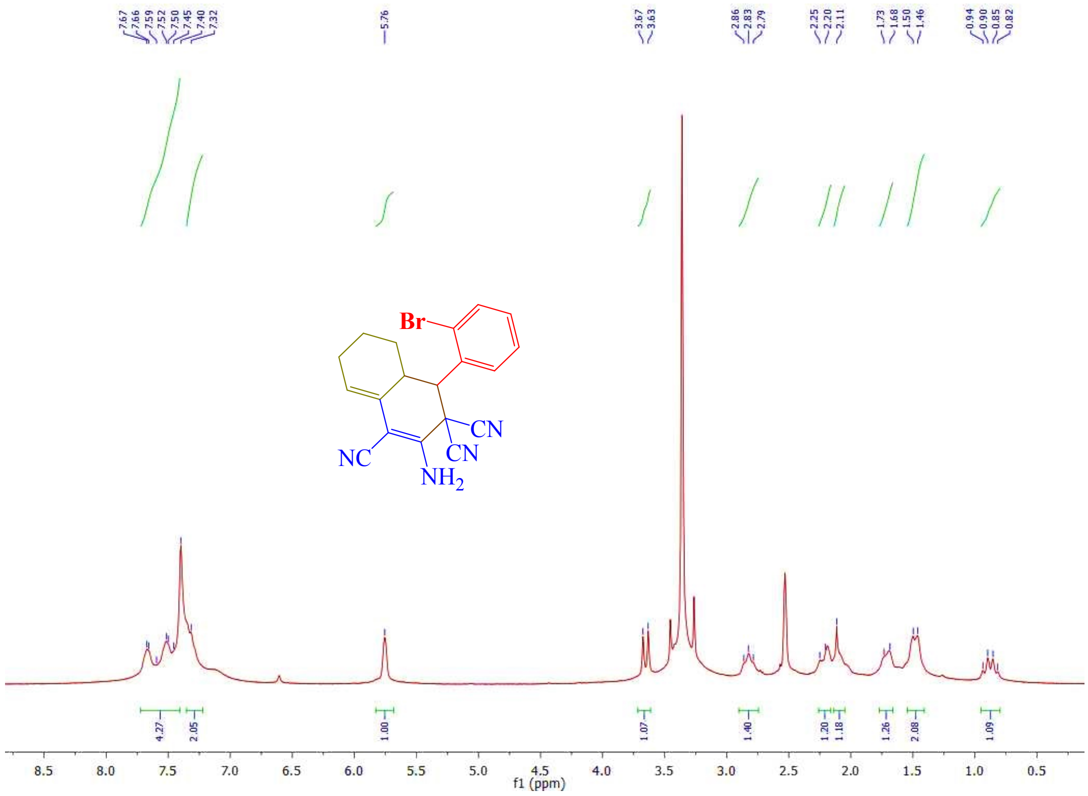

Fig. S21. ^{13}C NMR spectrum of 2-amino-4-(2-boromophenyl)-4a,5,6,7-tetrahydronaphthalene-1,3,3(4*H*)-tricarbonitrile (6l)

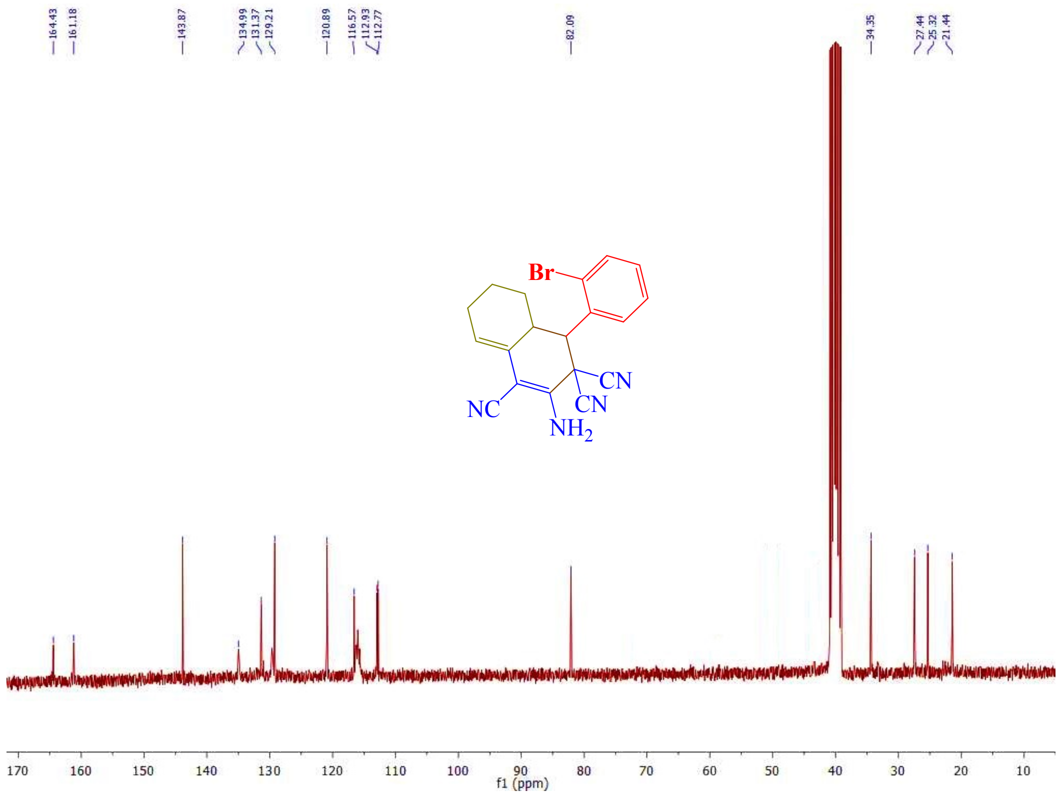

Fig. S22. FT-IR spectrum of 2-amino-4a,5,6,7-tetrahydro-4-p-tolylnaphthalene-1,3,3(*4H*)-tricarbonitrile (6m)

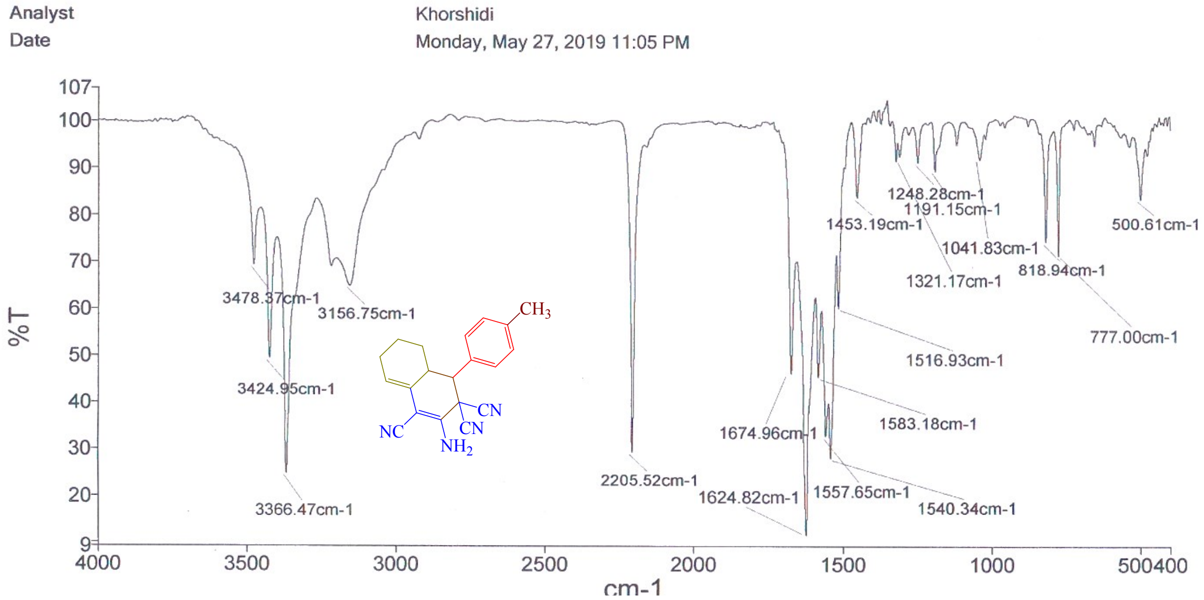

Fig. S23. ^{1}H NMR spectrum of 2-amino-4a,5,6,7-tetrahydro-4-p-tolylnaphthalene-1,3,3(*4H*)-tricarbonitrile (6m)

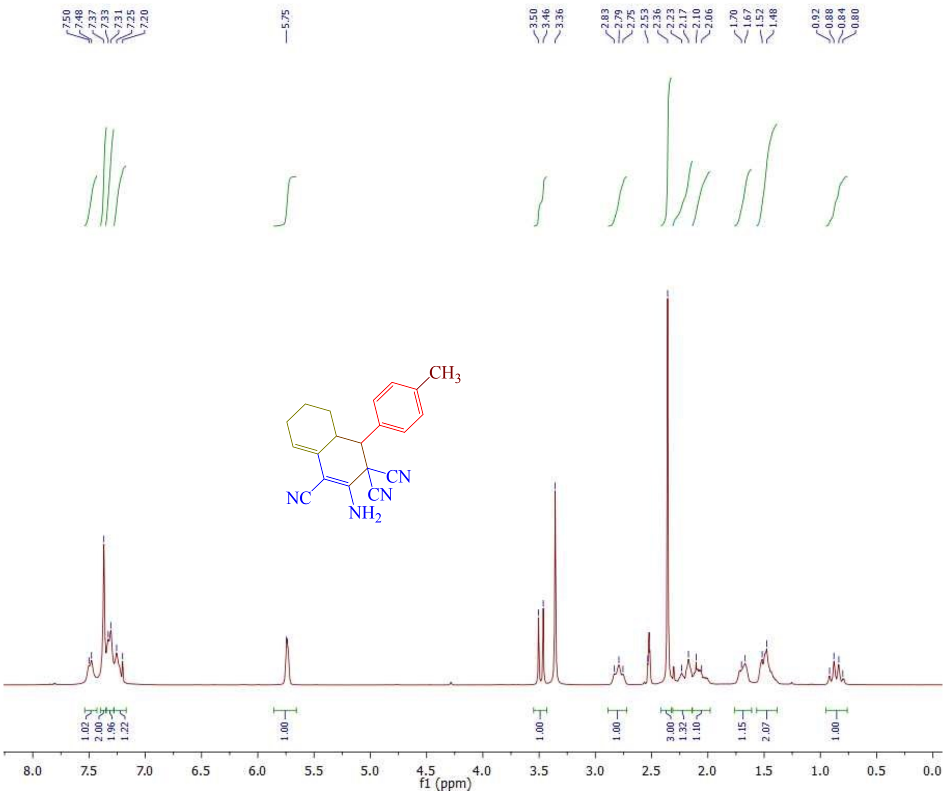
7.50 7.48 7.37 7.33 7.31 7.25 7.20
5.75
3.50 3.46 3.36
2.83 2.79 2.75 2.53 2.36 2.23 2.17 2.10 2.06
1.70 1.67 1.52 1.48
0.92 0.88 0.84 0.80
CH3
CN
CN
NC
NH2
1.02 2.00 1.96 1.22
1.00
1.00
1.00
3.00 1.32 1.10
1.15 2.07
1.00
8.0 7.5 7.0 6.5 6.0 5.5 5.0 4.5 4.0 3.5 3.0 2.5 2.0 1.5 1.0 0.5 0.0
f1 (ppm)

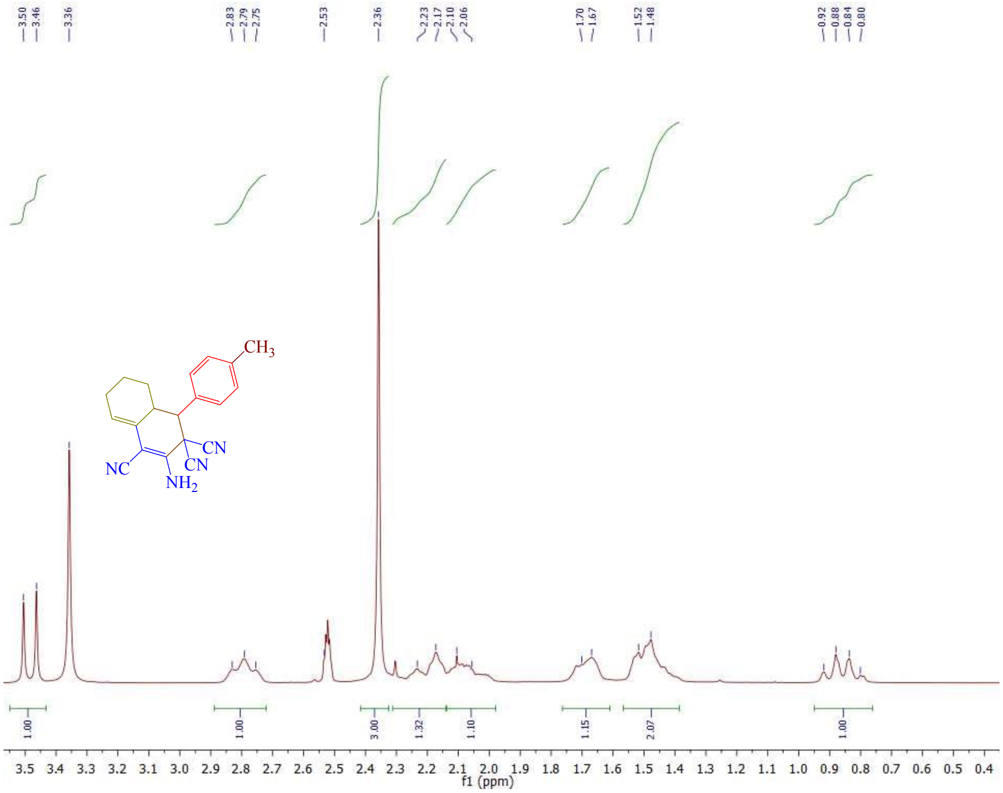
3.50 3.46 3.36
2.83 2.79 2.75
2.53
2.36
2.23 2.17 2.10 2.06
1.70 1.67
1.52 1.48
0.92 0.88 0.84 0.80
CH3
CN
CN
NC
NH2
1.00
1.00
3.00 1.32 1.10
1.15 2.07
1.00
3.5 3.4 3.3 3.2 3.1 3.0 2.9 2.8 2.7 2.6 2.5 2.4 2.3 2.2 2.1 2.0 1.9 1.8 1.7 1.6 1.5 1.4 1.3 1.2 1.1 1.0 0.9 0.8 0.7 0.6 0.5 0.4
f1 (ppm)

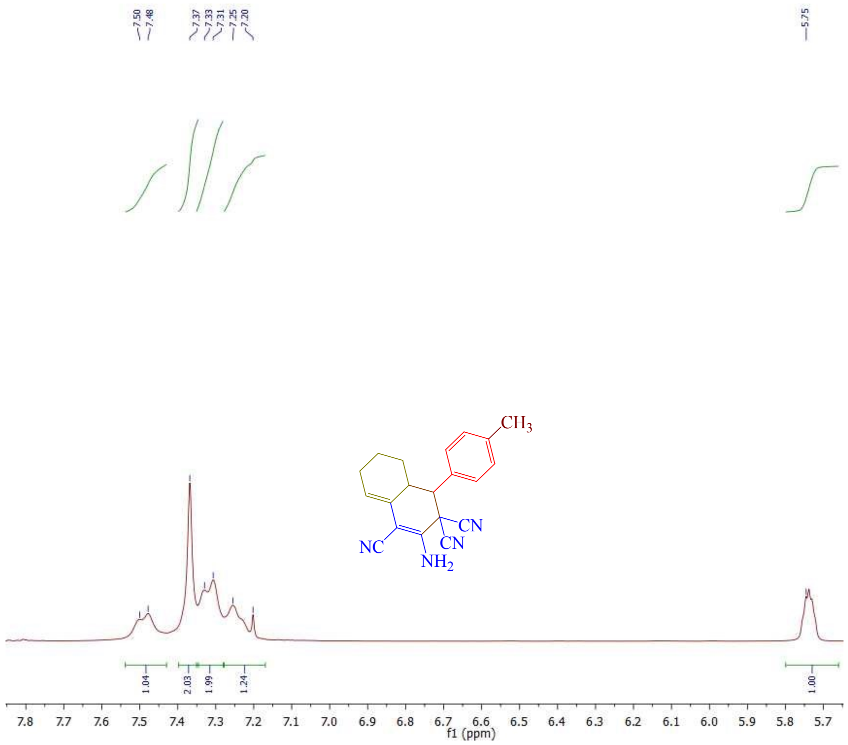
7.50
7.48
7.37
7.33
7.31
7.25
7.20
5.75
CH3
NC
CN
CN
NH2
1.04
2.03
1.99
1.24
1.00
7.8 7.7 7.6 7.5 7.4 7.3 7.2 7.1 7.0 6.9 6.8 6.7 6.6 6.5 6.4 6.3 6.2 6.1 6.0 5.9 5.8 5.7
f1 (ppm)

Fig. S24. ^{13}C NMR spectrum of 2-amino-4a,5,6,7-tetrahydro-4-p-tolylnaphthalene-1,3,3(*4H*)-tricarbonitrile (6m)

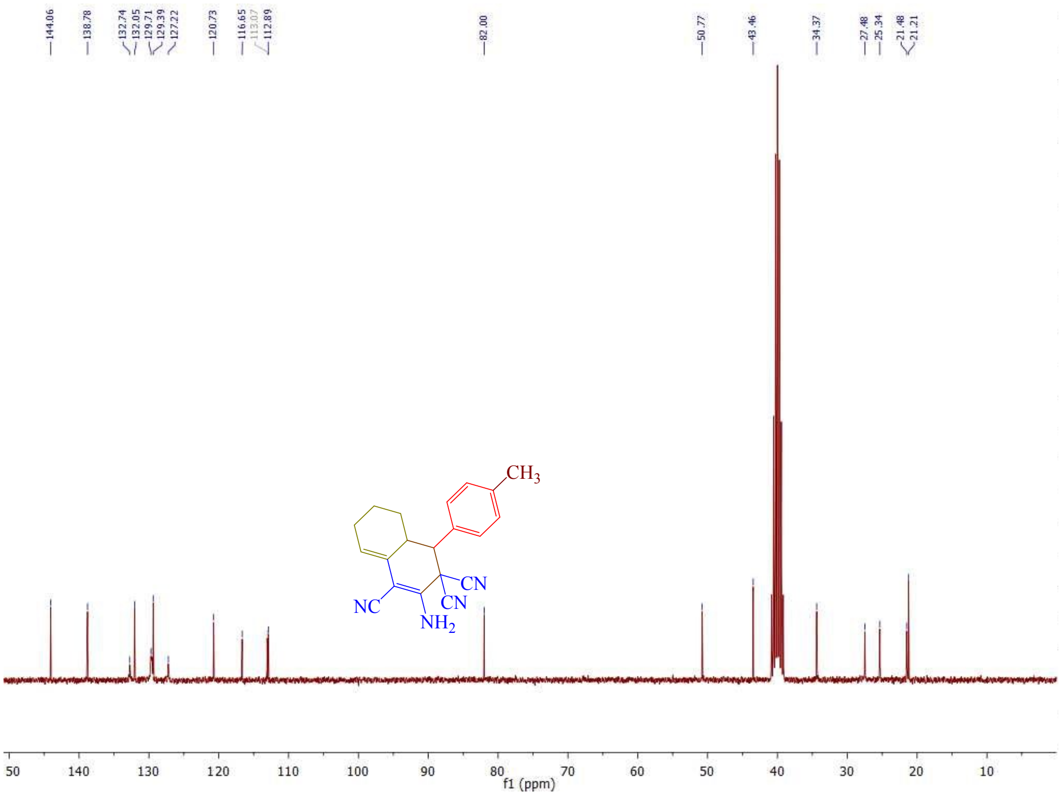

Fig. S25. FT-IR spectrum of 2-amino-4-(4-methoxyphenyl)-4a,5,6,7-tetrahydronaphthalene-1,3,3(4*H*)-tricarbonitrile (6n)

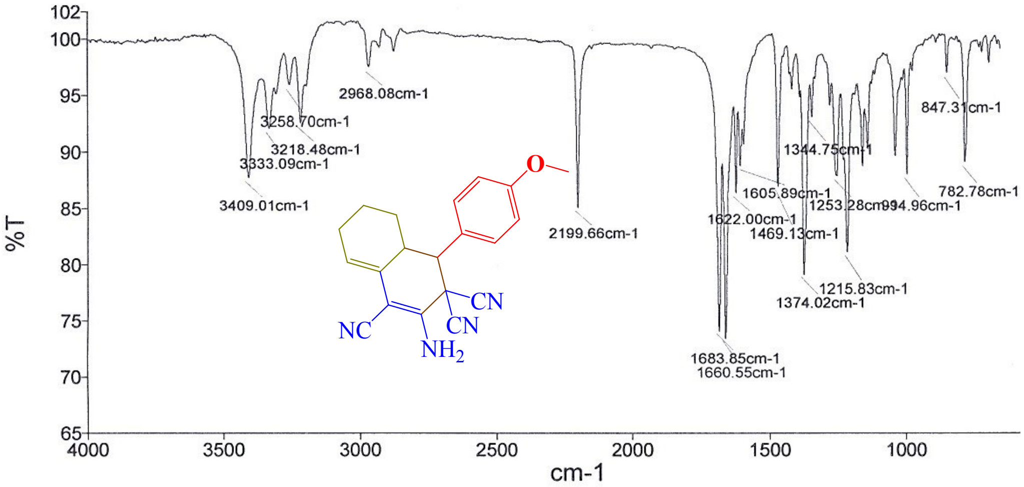

Fig. S26. ^{1}H NMR spectrum of 2-amino-4-(4-methoxyphenyl)-4a,5,6,7-tetrahydronaphthalene-1,3,3(4*H*)-tricarbonitrile (6n)

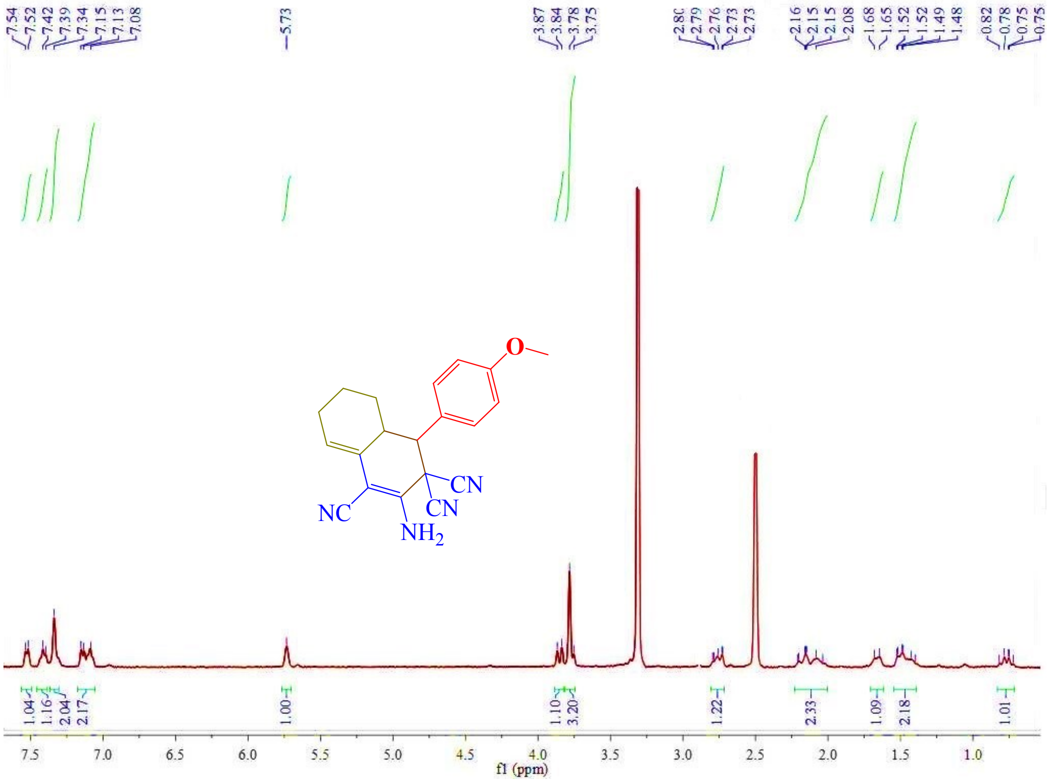

Fig. S27. ^{13}C NMR spectrum of 2-amino-4-(4-methoxyphenyl)-4a,5,6,7-tetrahydronaphthalene-1,3,3(4*H*)-tricarbonitrile (6n)

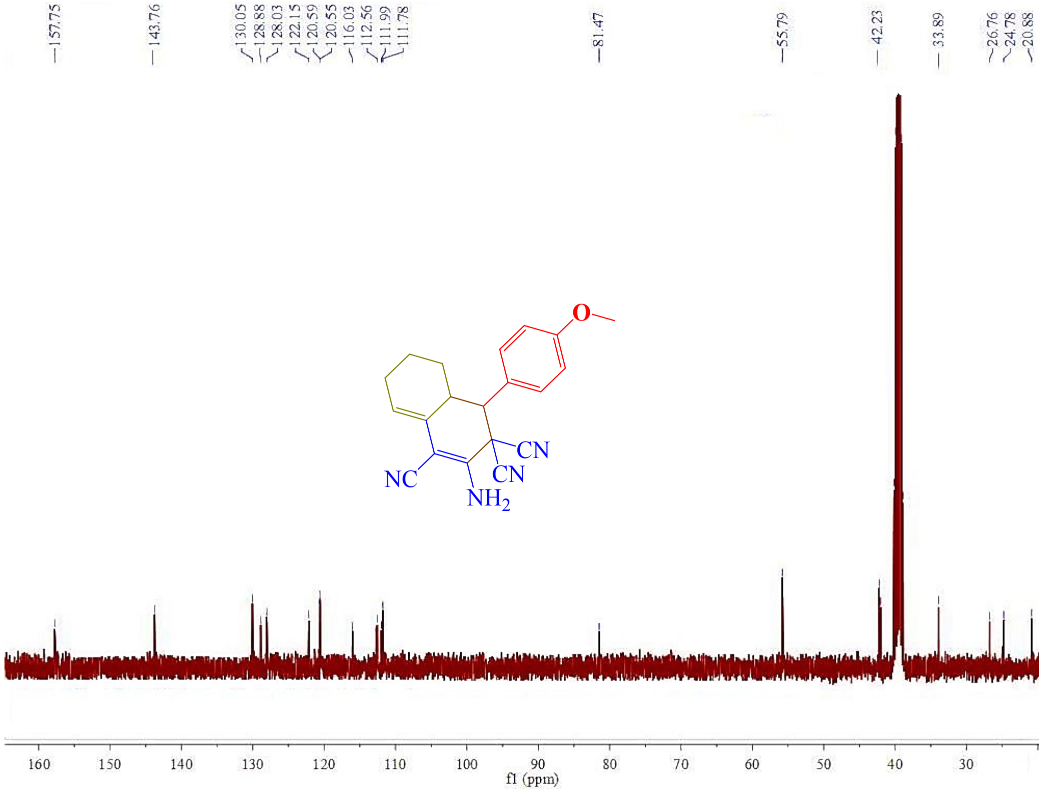

Fig. S28. FT-IR spectrum of 2-amino-4-(3,4-Dimethoxyphenyl)-4a,5,6,7-tetrahydronaphthalene-1,3,3(4*H*)-tricarbonitrile (6p)

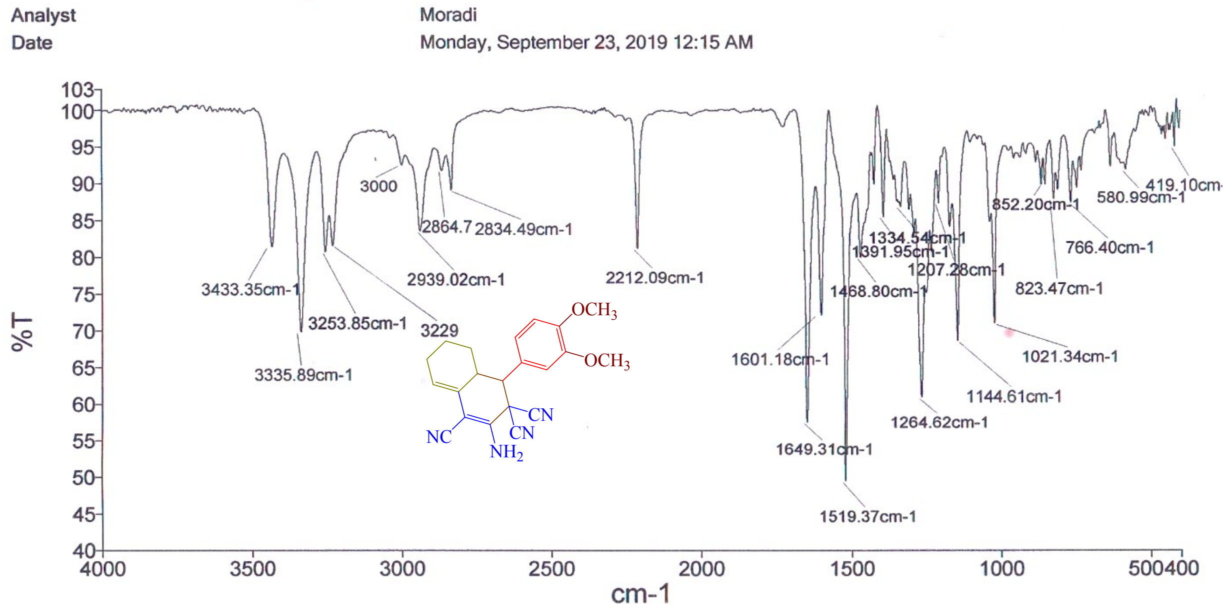

Fig. S29. ^{1}H NMR spectrum of 2-amino-4-(3,4-Dimethoxyphenyl)-4a,5,6,7-tetrahydronaphthalene-1,3,3(4*H*)-tricarbonitrile (6p)

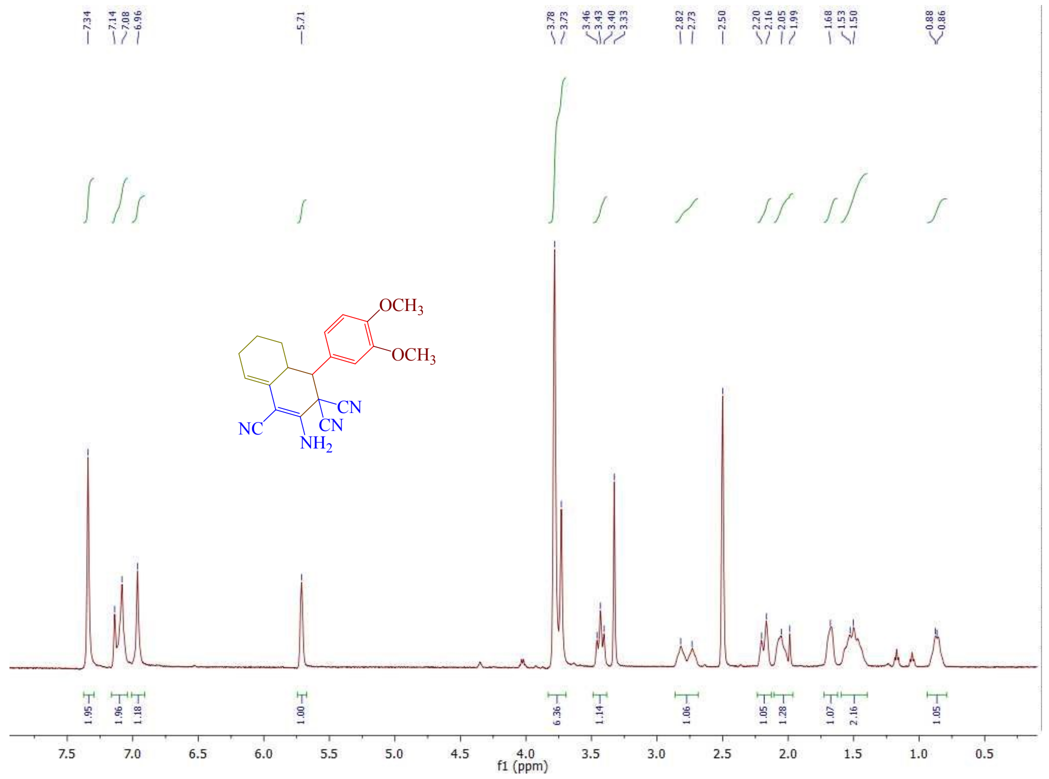

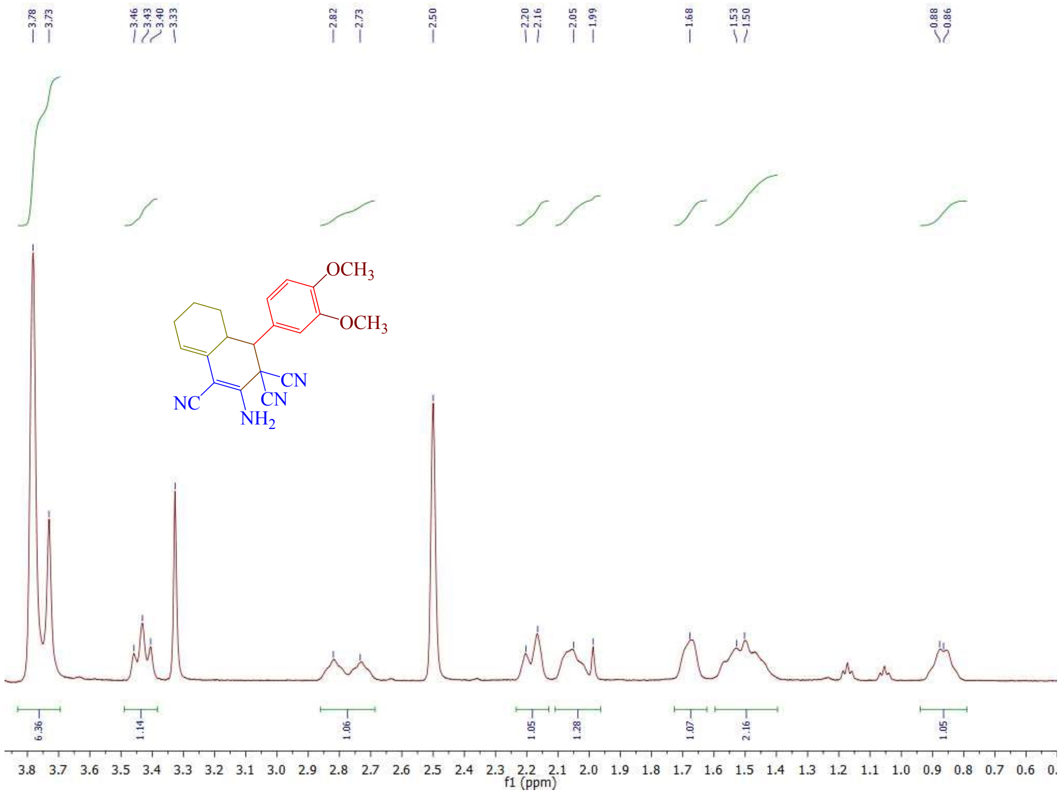

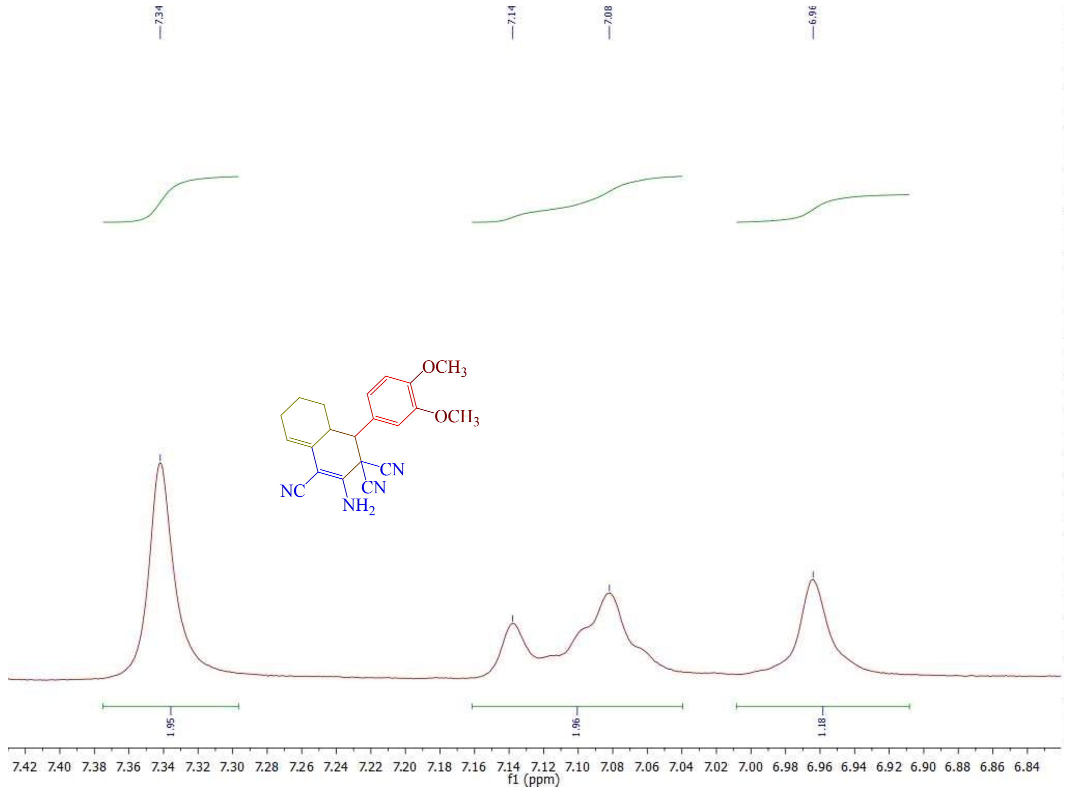

7.34
7.14
7.08
6.96
OCH3
OCH3
NC
CN
CN
NH2
1.95
1.96
1.18
7.42 7.40 7.38 7.36 7.34 7.32 7.30 7.28 7.26 7.24 7.22 7.20 7.18 7.16 7.14 7.12 7.10 7.08 7.06 7.04 7.02 7.00 6.98 6.96 6.94 6.92 6.90 6.88 6.86 6.84
f1 (ppm)

Fig. S30. ^{13}C NMR spectrum of 2-amino-4-(3,4-Dimethoxyphenyl)-4a,5,6,7-tetrahydronaphthalene-1,3,3(4*H*)-tricarbonitrile (6p)

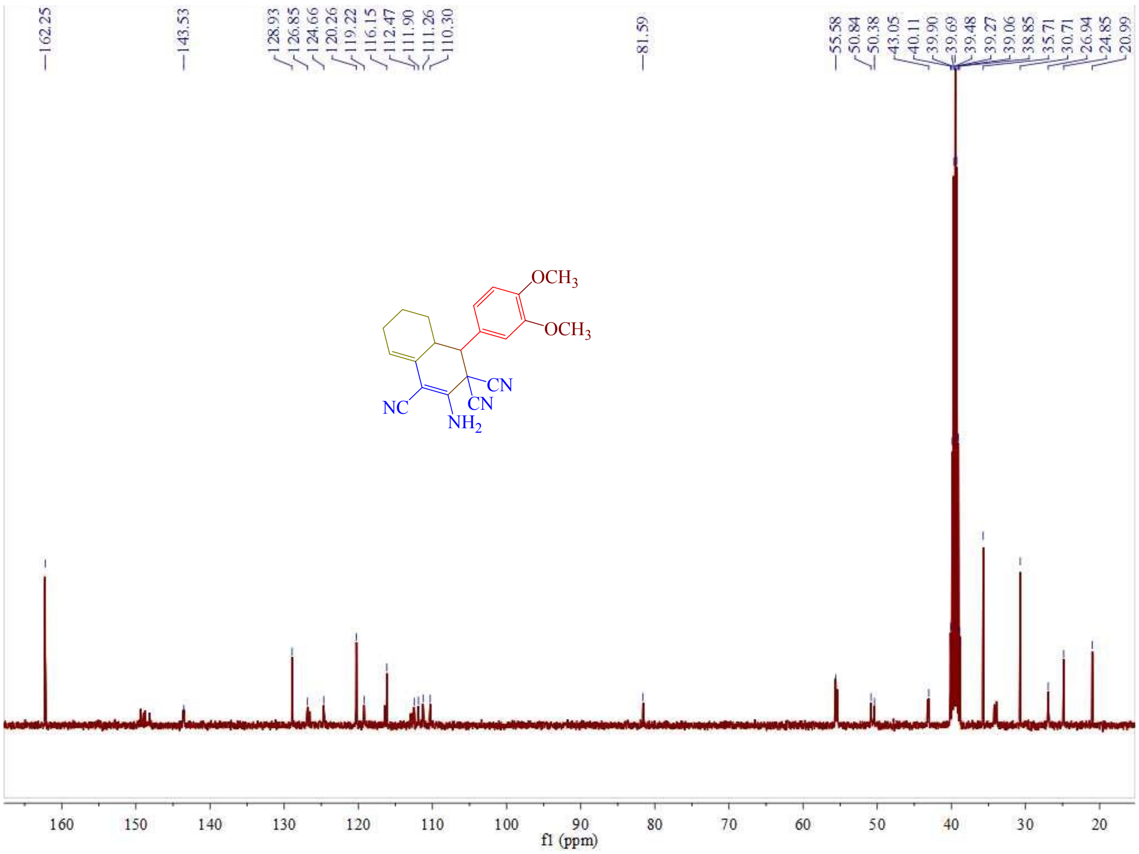

Fig. S31. FT-IR spectrum of 2-amino-4-(4-methoxyphenyl)-5,6,7,8-tetrahydroquinoline-3-carbonitrile (8b)

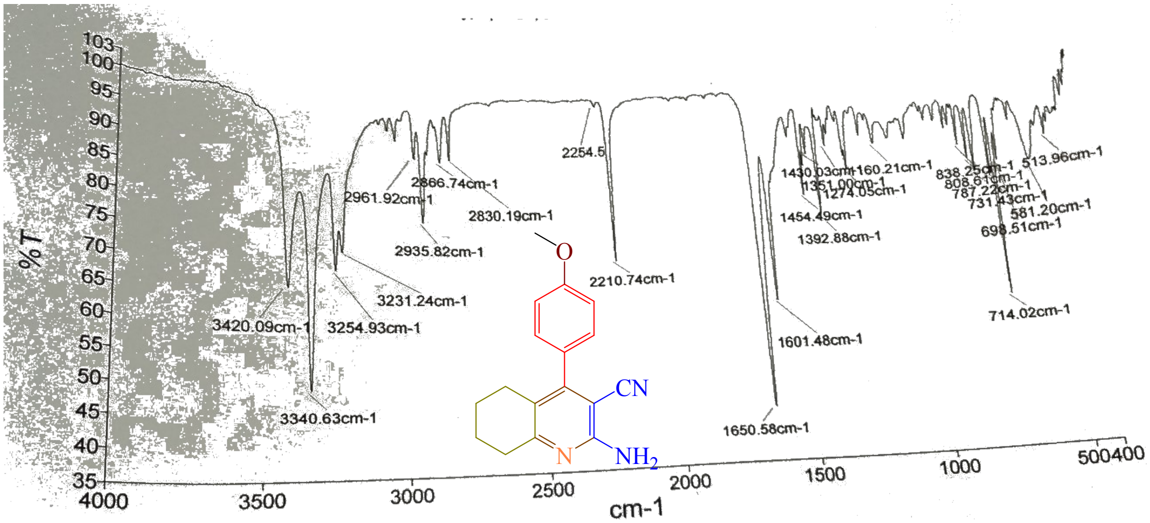

Fig. S32. ^{1}H NMR spectrum of 2-amino-4-(4- methoxyphenyl)-5,6,7,8-tetrahydroquinoline-3-carbonitrile (8b)

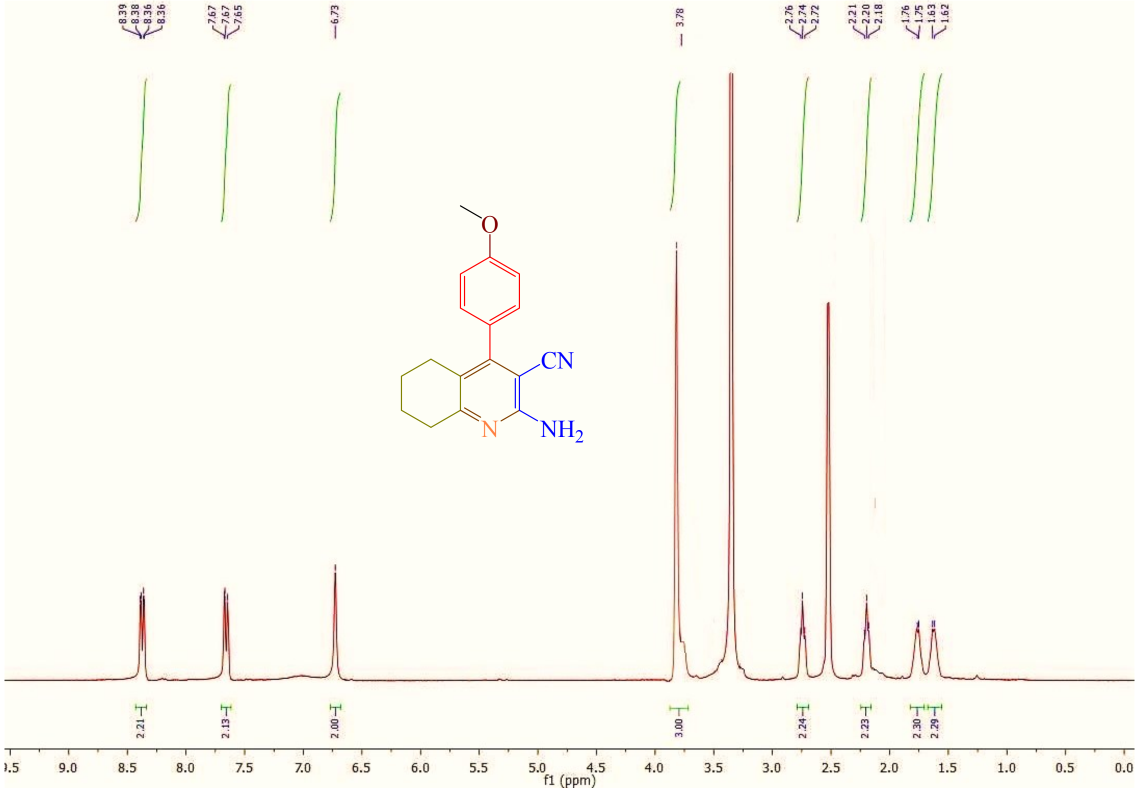

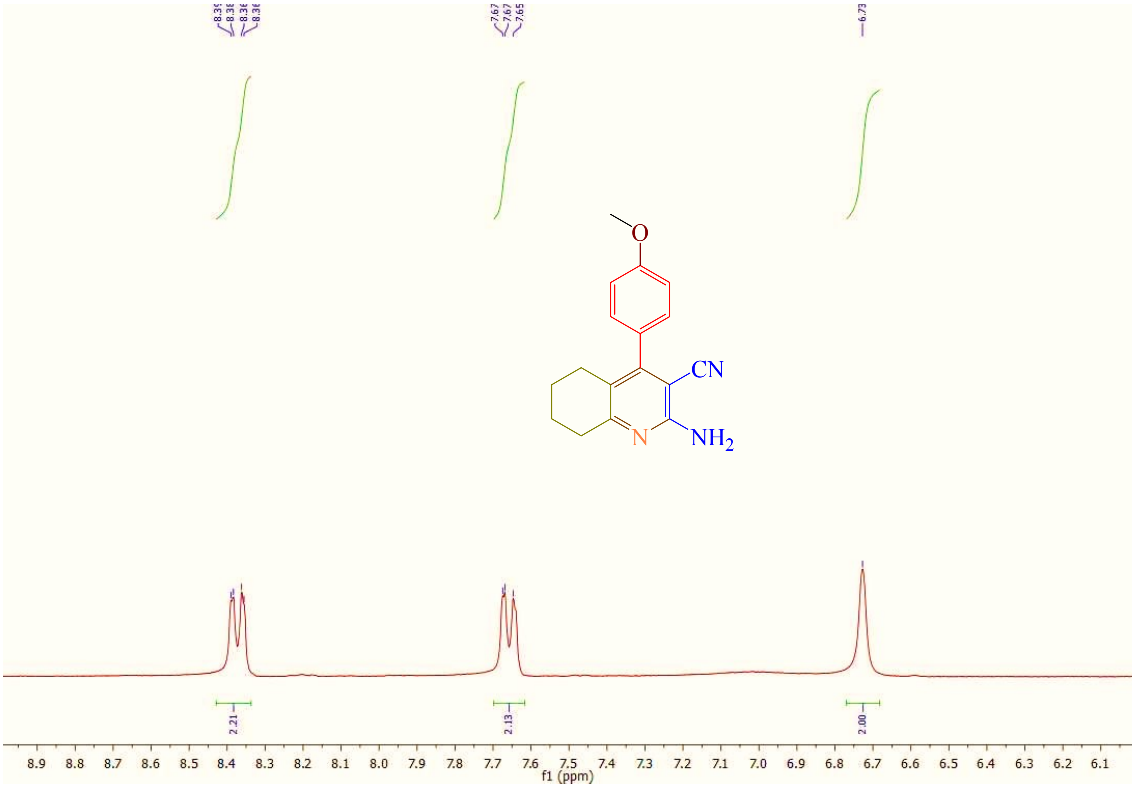

Fig. S33. ^{13}C NMR spectrum of 2-amino-4-(4- methoxyphenyl)-5,6,7,8-tetrahydroquinoline-3-carbonitrile (8b)

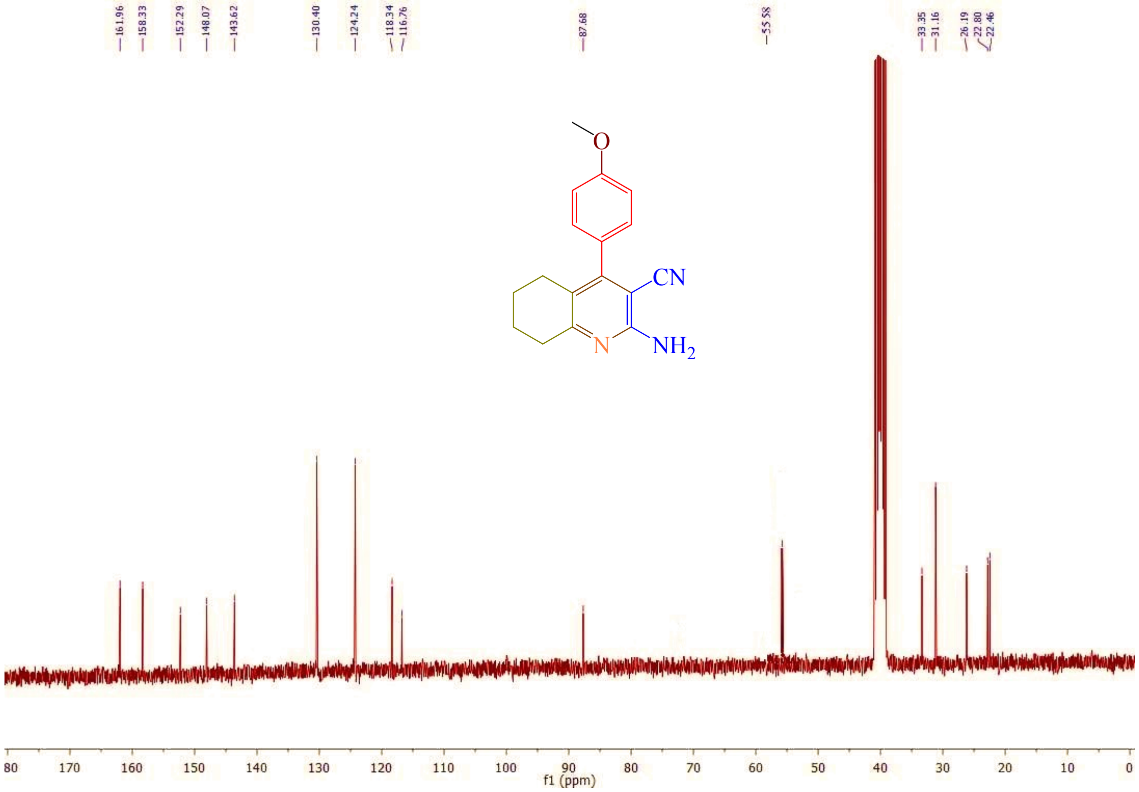

Fig. S34. FT-IR spectrum of 2-amino-4-(4-chlorophenyl)-5,6,7,8-tetrahydroquinoline-3-carbonitrile (8d)

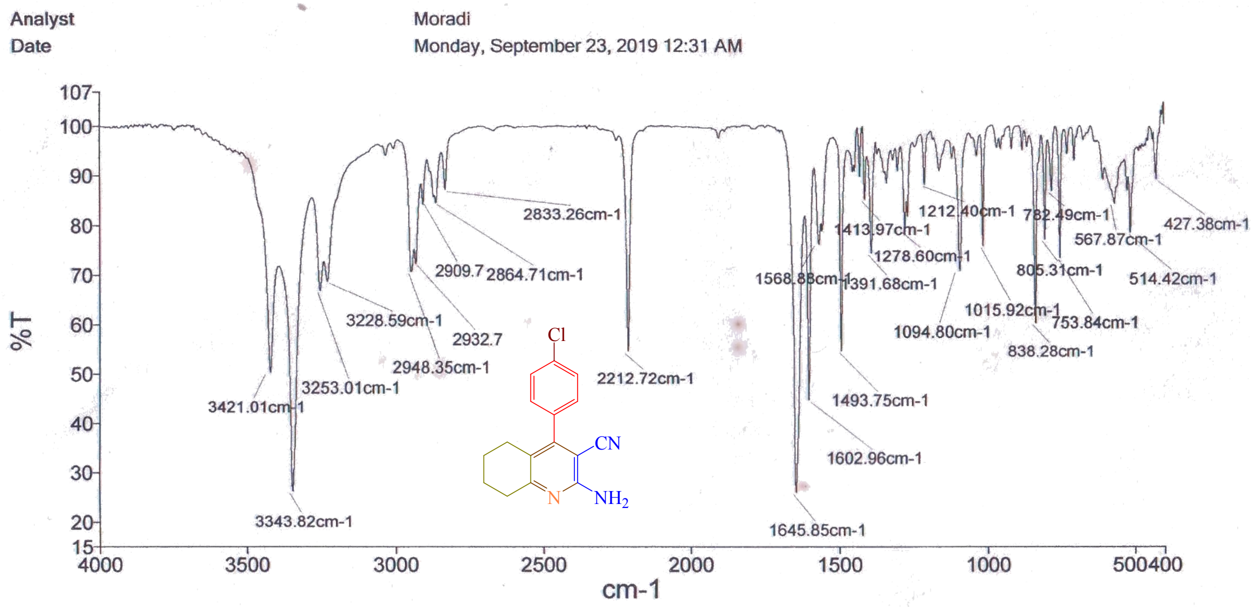

Fig. S35. ^{1}H NMR spectrum of 2-amino-4-(4-chlorophenyl)-5,6,7,8-tetrahydroquinoline-3-carbonitrile (8d)

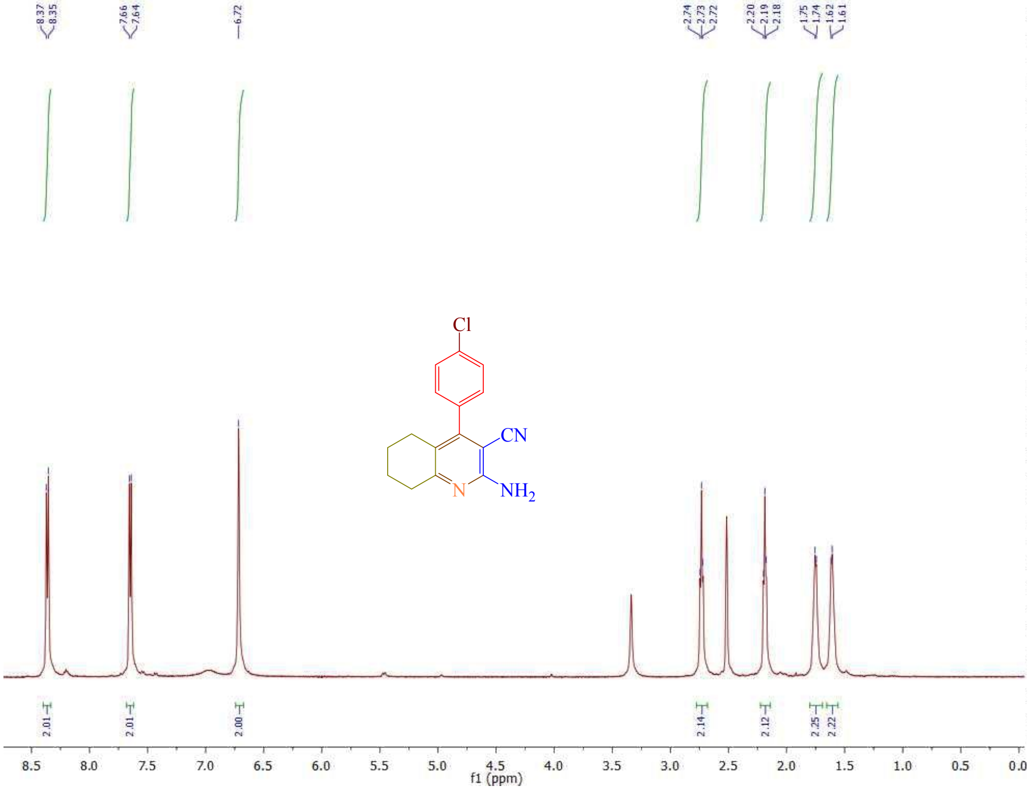

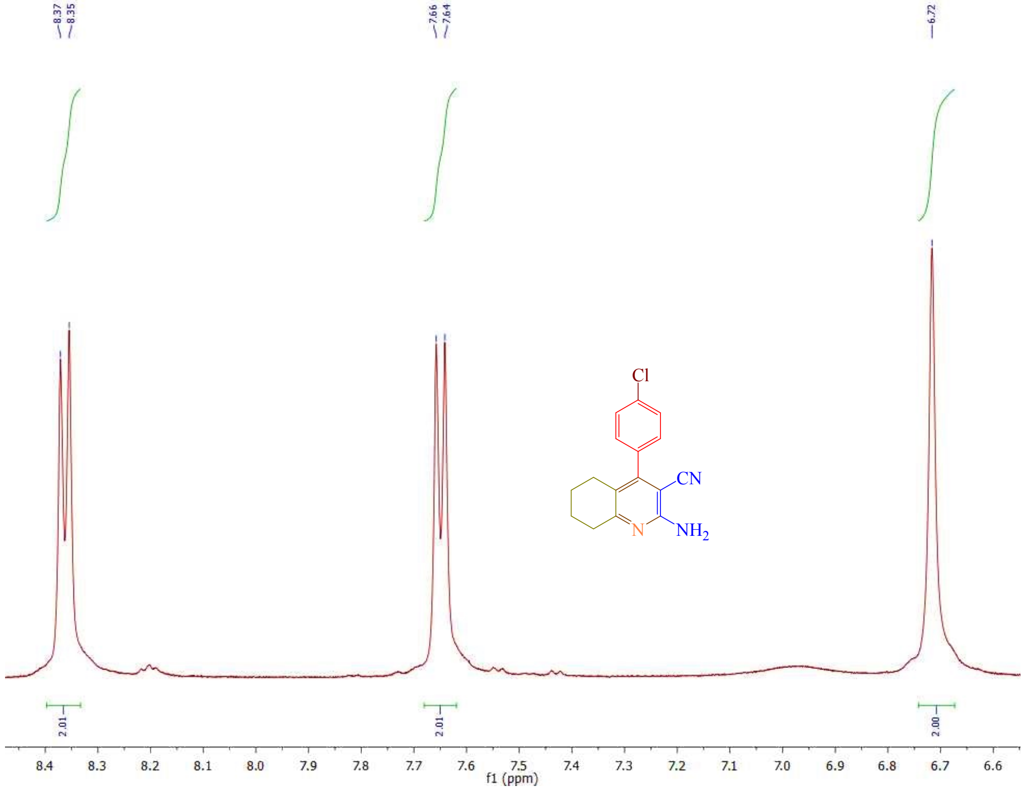

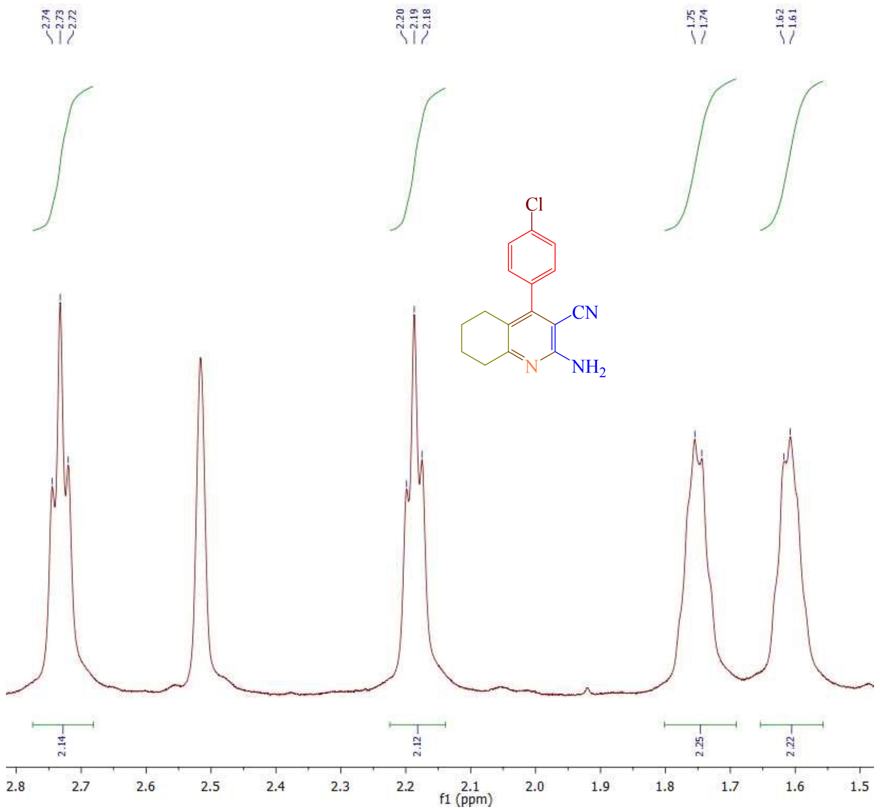

Fig. S36. ^{13}C NMR spectrum of 2-amino-4-(4-chlorophenyl)-5,6,7,8-tetrahydroquinoline-3-carbonitrile (8d)

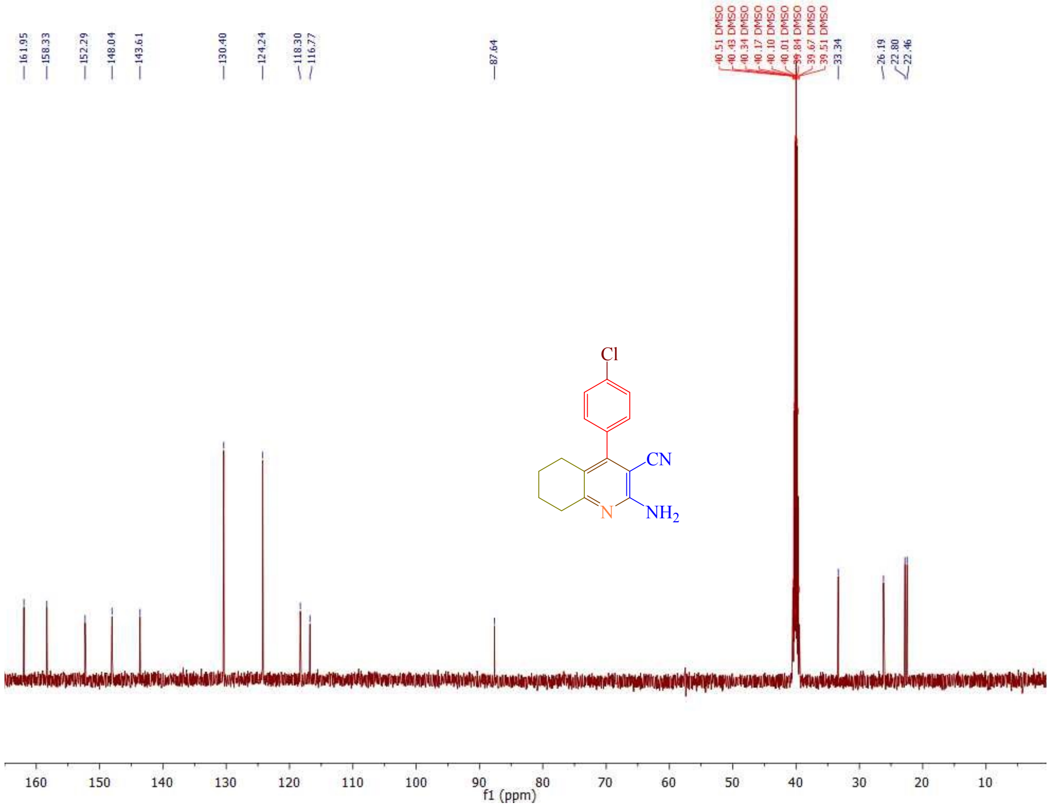

Fig. S37. FT-IR spectrum of 2-amino-4-(4-bromophenyl)-5,6,7,8-tetrahydroquinoline-3-carbonitrile (8e)

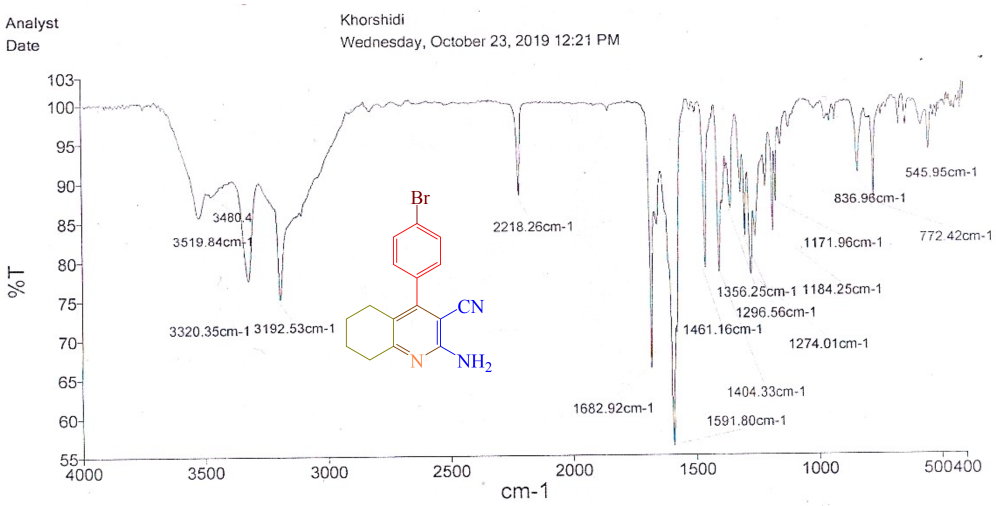

Fig S38. ^{1}H NMR spectrum of 2-amino-4-(4-bromophenyl)-5,6,7,8-tetrahydroquinoline-3-carbonitrile (8e)

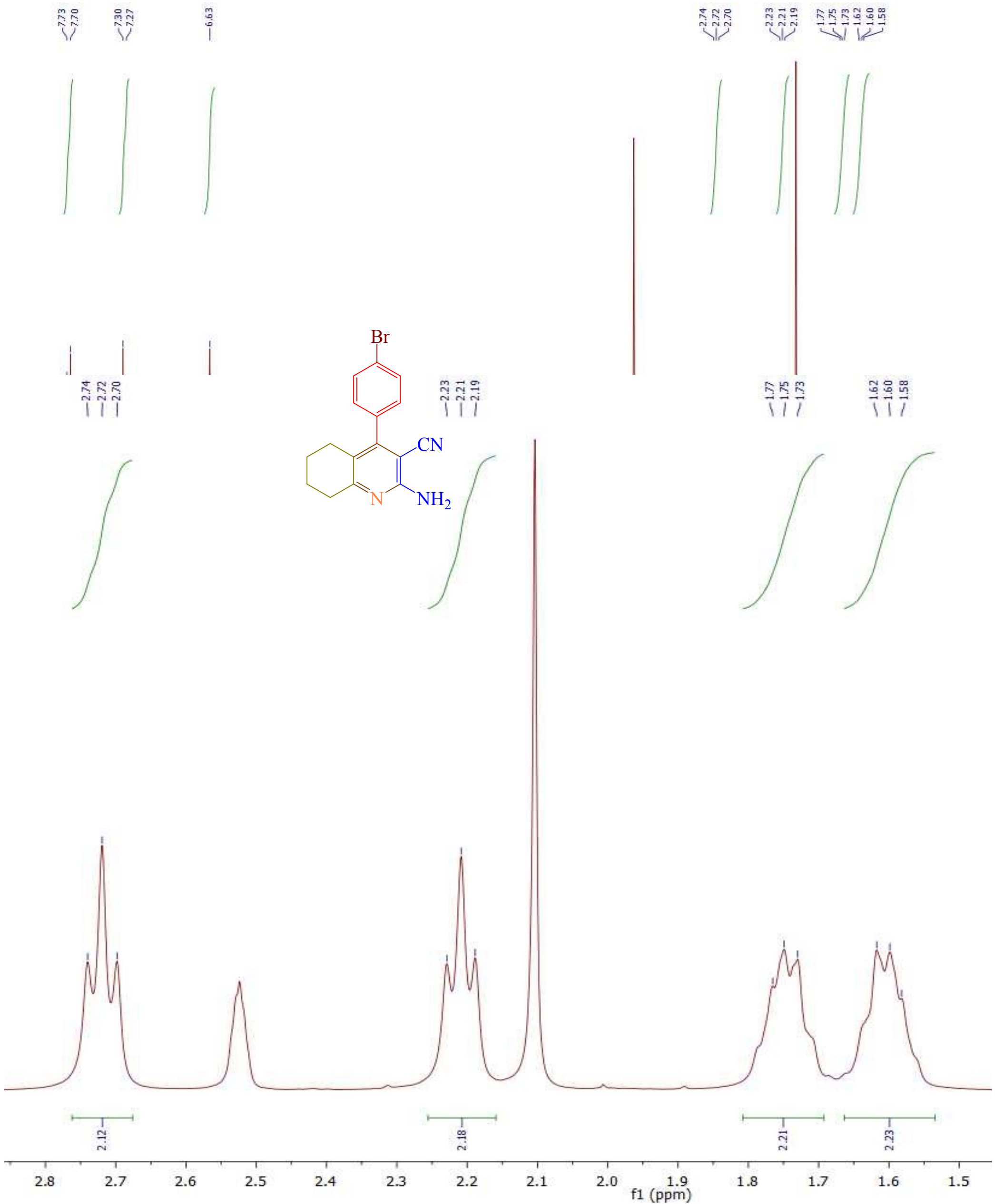

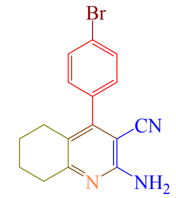

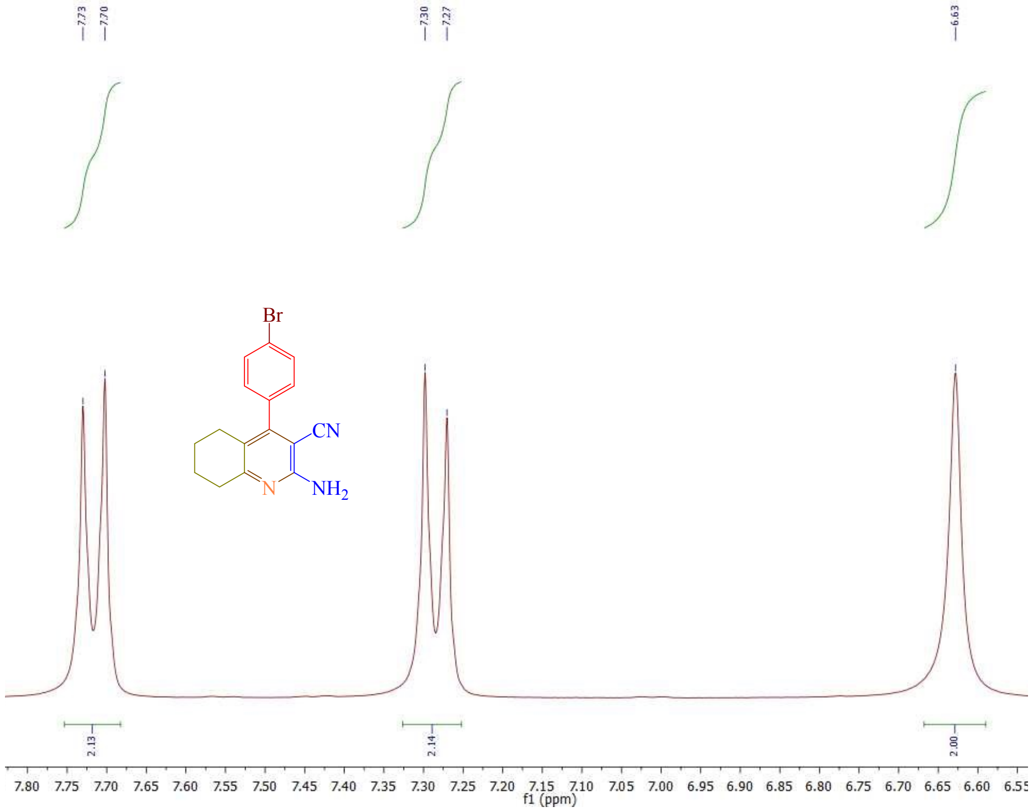

Fig. S39. ^{13}C NMR spectrum of 2-amino-4-(4-bromophenyl)-5,6,7,8-tetrahydroquinoline-3-carbonitrile (8e)

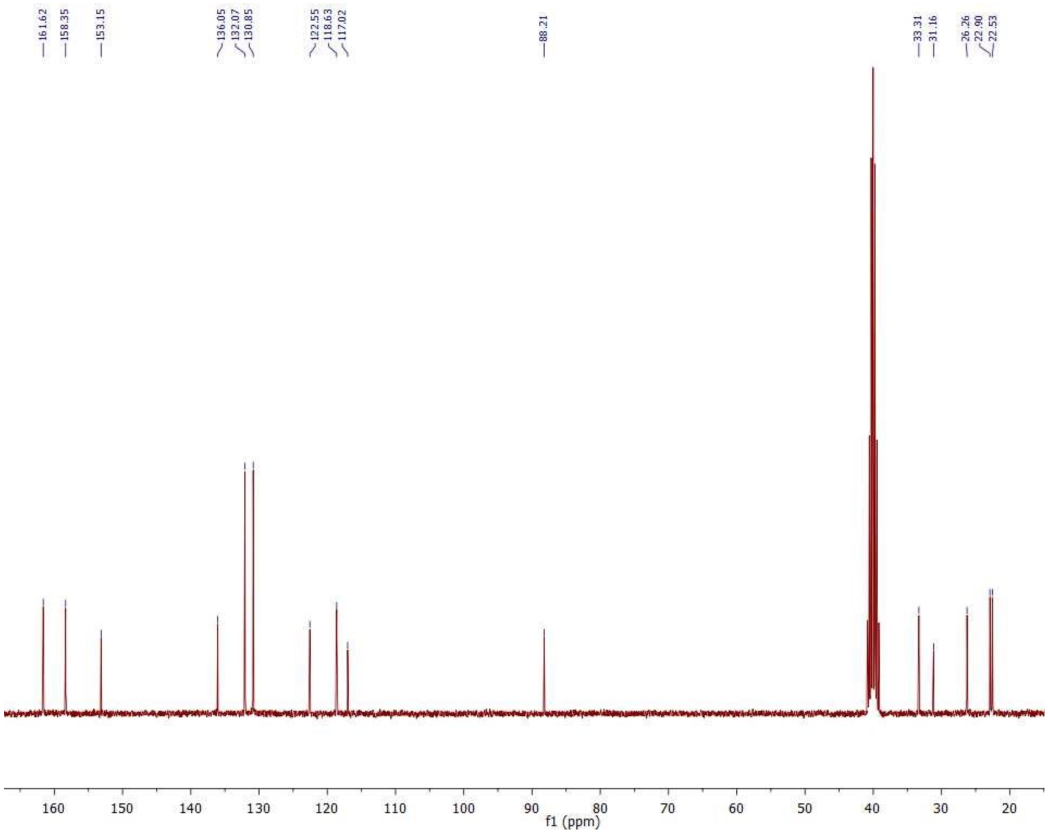

Fig. S40. FT-IR spectrum of 2-amino-4-(thiophen-2-yl)-5,6,7,8-tetrahydroquinoline-3-carbonitrile (8f)

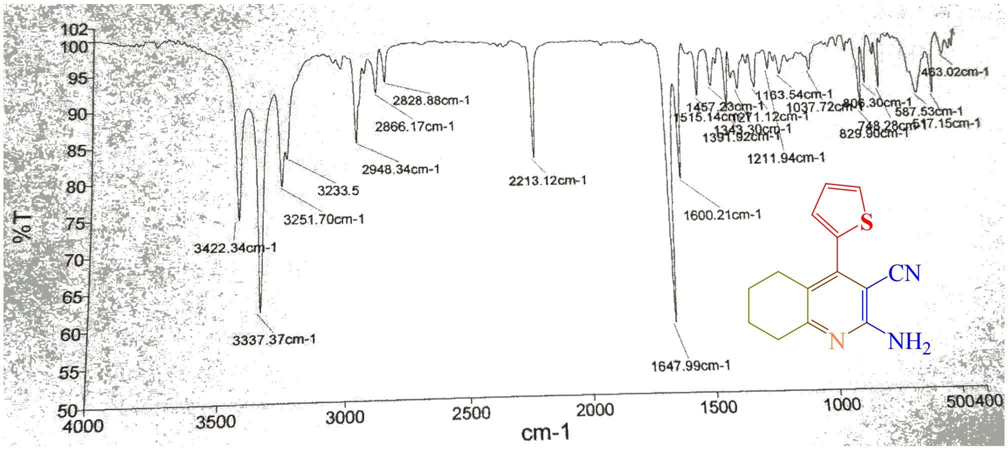

Fig. S41. ^{1}H NMR spectrum of 2-amino-4-(thiophen-2-yl)-5,6,7,8-tetrahydroquinoline-3-carbonitrile (8f)

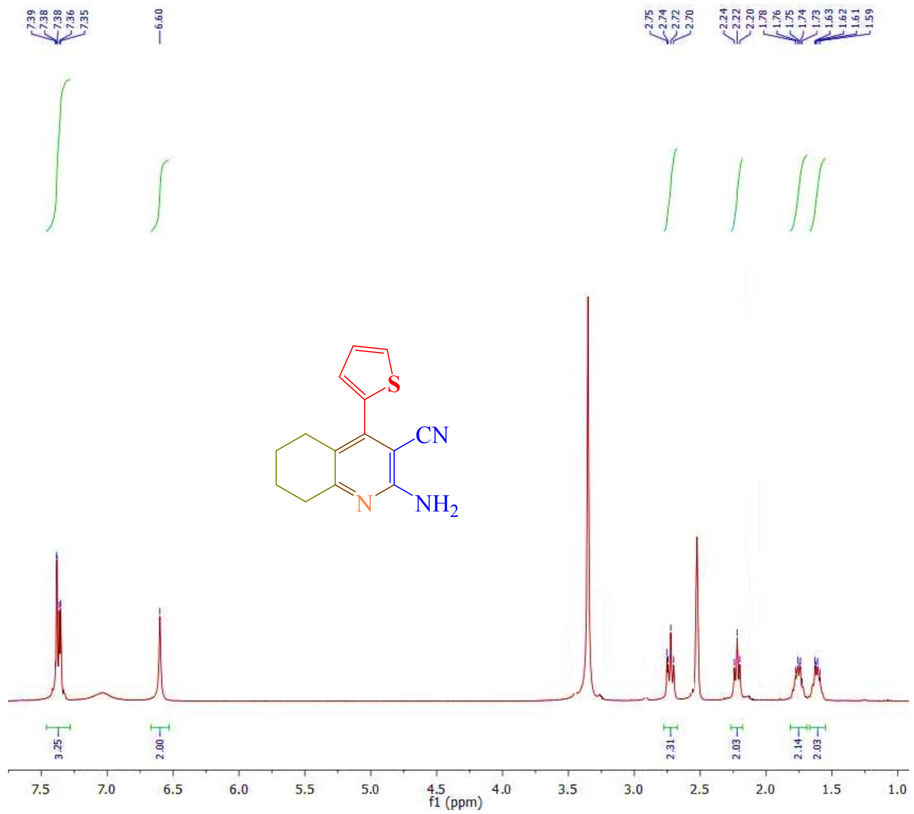

Fig. S42. ^{13}C NMR spectrum of 2-amino-4-(thiophen-2-yl)-5,6,7,8-tetrahydroquinoline-3-carbonitrile (8f)

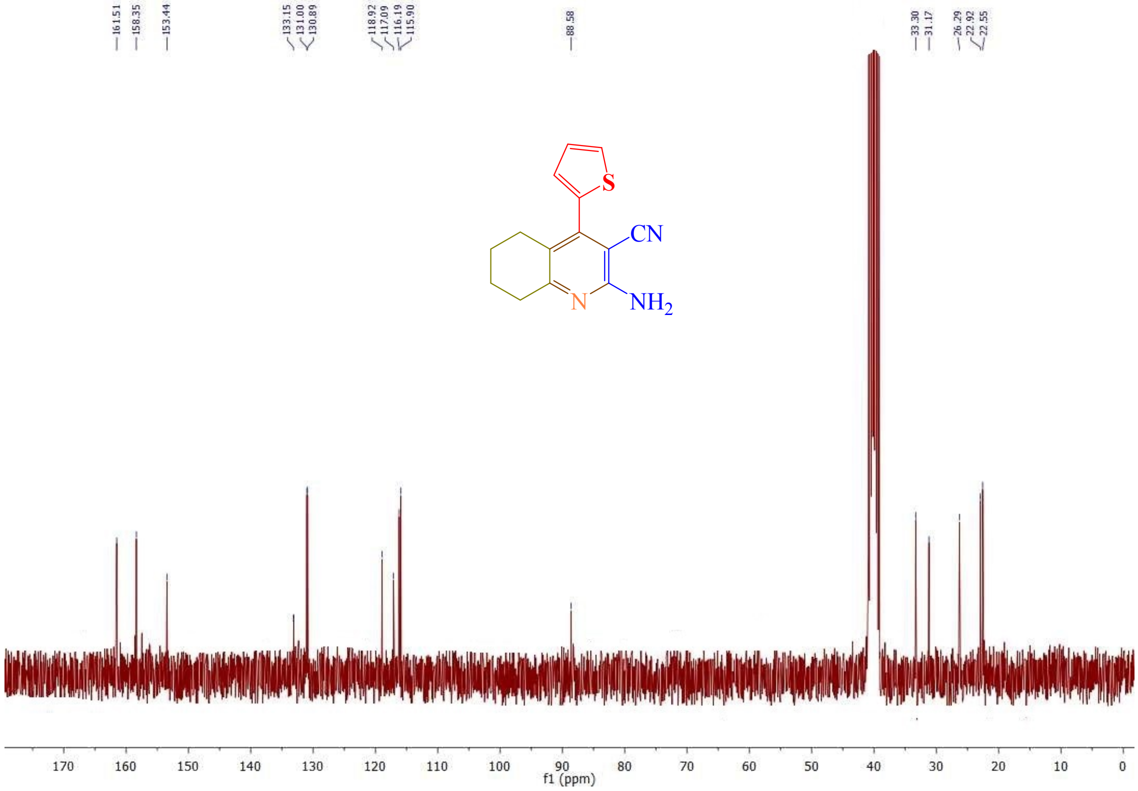

Supplement: RA-010-D0RA02172E-s001 [file RA-010-D0RA02172E-s001.pdf]
